# Immune-mediated myogenesis and acetylcholine receptor clustering promote a slow disease progression in ALS mouse models

**DOI:** 10.1186/s41232-023-00270-w

**Published:** 2023-03-09

**Authors:** Cassandra Margotta, Paola Fabbrizio, Marco Ceccanti, Chiara Cambieri, Gabriele Ruffolo, Jessica D’Agostino, Maria Chiara Trolese, Pierangelo Cifelli, Veronica Alfano, Christian Laurini, Silvia Scaricamazza, Alberto Ferri, Gianni Sorarù, Eleonora Palma, Maurizio Inghilleri, Caterina Bendotti, Giovanni Nardo

**Affiliations:** 1 Laboratory of Molecular Neurobiology, Department of Neuroscience, Istituto di Ricerche Farmacologiche Mario Negri IRCCS, Via Mario Negri 2, 20156 Milan, Italy; 2 Department of Human Neurosciences, Rare Neuromuscular Diseases Centre, Sapienza University of Rome, 00185 Rome, Italy; 3 Laboratory Affiliated to Istituto Pasteur Italia, Department of Physiology and Pharmacology, Sapienza University of Rome, 00185 Rome, Italy; 4 IRCCS San Raffaele Roma, 00163 Rome, Italy; 5 Department of Applied Clinical and Biotechnological Sciences, University of L’Aquila, 67100 L’Aquila, Italy; 6 IRCCS Fondazione Santa Lucia, Rome, Italy; 7 Institute of Translational Pharmacology (IFT-CNR), Rome, Italy; 8 Department of Neuroscience, Azienda Ospedaliera di Padova, Via Giustiniani 2, 35128 Padua, Italy

**Keywords:** Amyotrophic lateral sclerosis, Mouse model, ACh receptor, Skeletal muscle, Macrophages, Myogenesis, Satellite cells

## Abstract

**Background:**

Amyotrophic lateral sclerosis (ALS) is a heterogeneous disease in terms of onset and progression rate. This may account for therapeutic clinical trial failure. Transgenic SOD1G93A mice on C57 or 129Sv background have a slow and fast disease progression rate, mimicking the variability observed in patients.

Based on evidence inferring the active influence of skeletal muscle on ALS pathogenesis, we explored whether dysregulation in hindlimb skeletal muscle reflects the phenotypic difference between the two mouse models.

**Methods:**

Ex vivo immunohistochemical, biochemical, and biomolecular methodologies, together with in vivo electrophysiology and in vitro approaches on primary cells, were used to afford a comparative and longitudinal analysis of *gastrocnemius medialis* between fast- and slow-progressing ALS mice.

**Results:**

We reported that slow-progressing mice counteracted muscle denervation atrophy by increasing acetylcholine receptor clustering, enhancing evoked currents, and preserving compound muscle action potential. This matched with prompt and sustained myogenesis, likely triggered by an early inflammatory response switching the infiltrated macrophages towards a M2 pro-regenerative phenotype. Conversely, upon denervation, fast-progressing mice failed to promptly activate a compensatory muscle response, exhibiting a rapidly progressive deterioration of muscle force.

**Conclusions:**

Our findings further pinpoint the pivotal role of skeletal muscle in ALS, providing new insights into underestimated disease mechanisms occurring at the periphery and providing useful (diagnostic, prognostic, and mechanistic) information to facilitate the translation of cost-effective therapeutic strategies from the laboratory to the clinic.

**Supplementary Information:**

The online version contains supplementary material available at 10.1186/s41232-023-00270-w.

## Introduction

Amyotrophic lateral sclerosis (ALS) is a fatal neurodegenerative disease characterised by degenerative changes in upper and lower motor neurons (MNs). It is the most common adult MN disease, with an incidence of 2 per 100,000 and a prevalence of 5.4 per 100,000 individuals [1]. Symptoms typically occur in late mid-life and present as relentlessly progressive muscle atrophy and weakness. In most cases, the effects on respiratory muscles limit survival to 2–4 years after disease onset. Current treatment options are based on symptom management and respiratory support. Riluzole and edaravone are the only approved medications widely used, providing modest benefits only in some patients [2]. While most ALS cases are sporadic (sALS), about 15% are familial (fALS) and characterised mostly by autosomal dominant inheritance.

The clinical manifestations of sALS and fALS are indistinguishable, suggesting that different pathways converge, causing typical neuromuscular degeneration. Technological advances have contributed to our understanding of the genetic causes of fALS, with approximately 40–55% of cases accounted for by variants in known ALS-linked genes. Although more than 50 potentially causative or disease-modifying genes have been identified, pathogenic modifications in SOD1, TARDBP, FUS, and C9ORF72 occur most frequently [3]. However, genetic advancements have helped explain only a fraction of sALS cases: the etiology remains unexplained in over 90% of patients.

The multisystemic nature of ALS pathology, which distinctively encompasses distant biological systems, makes it challenging to identify a proper therapeutic target. The limitations in disease understanding also pose challenges in developing more reliable, sensitive, and specific biomarkers of disease progression and prognostication. In keeping with this, ALS heterogeneity concerning age, site of onset, and disease progression is a decisive confounding factor in designing treatments and evaluating their efficacy [4, 5]. This variability is dependent on genetics, as documented by the high number of disease-associated gene variants, which may impact the age of onset [6]. Considerable disease heterogeneity also exists in ALS patients or mouse models carrying the same gene mutations (i.e. SOD1-linked ALS), surmising that genetic background-derived factors influence the disease manifestation [7, 8].

Evidence indicates that retrograde MN neurodegeneration could be integral to ALS pathogenesis in a targeted deprivation-type scenario where primary pathology in skeletal muscle could exacerbate MN loss [9]. This hypothesis is based on evidence that MN protection alone is insufficient to prevent peripheral axons and muscles from degenerating [10].

It is known that muscle atrophy associated with the dismantlement of the NMJs is one of the earliest events in ALS. Studies in transgenic ALS mice showed distal axon pathology and muscle denervation as early as 50 postnatal days when MNs do not show signs of degeneration [11]. Consistently, studies on muscles of mSOD1, mFUS, and mTDP43 mice showed early changes in neuromuscular transmission and the activation of the oxidative stress response system long before motor symptoms onset [12, 13]. Besides, considerable literature demonstrated mitochondrial defects, dysregulated muscle growth and development, and reduced muscle volume in mSOD1 mice from as early as 8 weeks of age, long before the disease onset [14, 15]. Muscle impairment is transmitted along the body by the secretion of anabolic signals that influence neuron survival, axonal growth, and synaptic connections. Indeed, it has been shown that the restricted expression of mSOD1 in MNs does not trigger ALS pathology [16]. Conversely, mSOD1 expression in skeletal muscle elicits muscle atrophy, decreases muscle strength, reduces spinal cord mass and MNs, and shortens lifespan [17]. This evidence indicates ALS as a distal axonopathy, where an intrinsic muscle pathology directly impacts MNs and, consequently, the quality of connections of motor units [18].

Hitherto, we lack relevant information about processes underlying muscle denervation atrophy in ALS. We recently demonstrated the pivotal role of the immune system in promoting and governing skeletal muscle innervation and regeneration and, thus, the speed of disease progression [19, 20]. Our evidence highlights the distinct contribution of the inflammatory response in the CNS compared to the periphery in ALS. Indeed, while the aberrant glial cell activation, T-cell infiltration, and the resulting release of proinflammatory molecules drive the neurodegeneration in the spinal cord, successful peripheral axon and muscle regeneration depend on the coordinated efforts of immune cells that, besides removing cellular debris, release factors that support the wound healing [19, 21]. This difference may explain the association between the peripheral nervous system (PNS) inflammation and the longer survival recently observed in ALS patients with SOD1 mutation [22].

We previously reported a remarkable difference in the disease onset and speed of symptoms progression in transgenic mice carrying the same amount of human SOD1G93A on different strains (C57BL/6JOlaHsd or 129/SvHsd) [8, 23]. Notably, SOD1G93A mice on C57BL/6JOlaHsd genetic background (slow-progressing mice; SP mice) showed a 3-week delay in the onset of symptoms and prolonged survival of about 8 weeks compared to SOD1G93A mice on 129SvHsd strain (fast-progressing mice; FP mice) [8]. Interestingly, we found that both mouse models showed a similar trend in spinal MN loss [23] but a difference in the rate of muscle wasting during the disease progression [24]. Mainly, FP mice showed abrupt muscle wasting at the disease onset, while SP mice had presymptomatic muscle atrophy that remained steady across the disease progression [24], suggesting the activation of compensatory mechanisms that could postpone symptom onset.

In this study, we found that early muscle denervation in SP mice is pivotal in eliciting an immune-mediated muscle pro-regenerative process associated with the generation of brand-new muscle fibres and increased fetal nicotinic acetylcholine receptors (AChRs) expression and clustering, which significantly enhance NMJ connections and function and delay the onset of muscle force deficit. These processes are faint in FP mice, which go towards rapid symptom onset upon muscle denervation followed by accelerated disease progression and MNs degeneration.

## Methods

### Animals

Female transgenic SOD1G93A mice on C57BL/6JOlaHsd (C57G93A) and 129S2/Sv (129SvG93A) genetic background, and their corresponding non-transgenic (NTG) female littermates, were used in this study [23, 25]. The animals have been housed under SPF (specific pathogen-free) standard conditions (22 ± 1 °C, 55 ± 10% relative humidity, and 12-h light/dark schedule), 3–4 per cage, with free access to food (standard pellet, Altromin, MT, Rieper) and water. Procedures involving animals and their care were conducted in conformity with the institutional guidelines of the Mario Negri Institute for Pharmacological Research, Milan, Italy, IRFMN, which are in compliance with national (D.lgs 26/2014; authorisation no. 783/2016-PR issued on August 8, 2016, by Ministry of Health) and Mario Negri institutional regulations and policies providing internal authorisation for persons conducting animal experiments (quality management system certificate—UNI EN ISO 9001:2008—reg. N° 6121); the NIH Guide for the Care and Use of Laboratory Animals (2011 edition); and EU directives and guidelines (EEC Council Directive 2010/63/UE). Animal studies were approved by the Mario Negri Institute Animal Care and Use Committee and by the Italian Ministerial Decree no. 246/2020-PR.

### Human skeletal muscle biopsies

Skeletal muscle biopsies were selected from the Biobank of the Neuromuscular Bank of Tissues at the University of Padua (Telethon Network of Genetic Biobanks; TNGB). Samples were frozen into the liquid phase of the isopentane, previously cooled in liquid nitrogen, for no more than 45 s. Frozen muscles were then stored at −80 °C until use. The monthly ALSFRS-R slope [progression rate to last visit (PRL) = 48-ALSFRS-R score at last visit/disease duration from the onset to the last visit] was used to define the rate of disease progression (*Δ*FS) of ALS patients [26]. Further clinical details on ALS patients from whom biopsies used in this study were derived are provided in Fabbrizio et al. [27].

### Nerve conduction study and electromyography (EMG)

Female mice 12 belonging to C57BL/6JOlaHsd (7 G93A, 5NTG), 10 belonging to 129S2/Sv (6 G93A, 4 NTG) strain were anaesthetised with 2.5-3% isoflurane during the procedure. The anaesthetised mice were stimulated with supramaximal monophasic square wave via two monopolar subdermal needles with the cathode near the ischiatic nerve and recording bilaterally from gastrocnemius muscles through a belly-tendon montage. A semiquantitative electromyographic evaluation of the gastrocnemius denervation pattern was performed bilaterally. Averaged data from the right and left sides at the presymptomatic stage was analysed.

Electromyography registrations were performed through a single-use coaxial electrode (Spes Medica, length 30 mm, diameter 0.35 mm, registration area 0.07 mm^2^) and analysed with Electromyographs System Plus Evolution MYOQUICK Matrix Line (Micromed, Mogliano Veneto [TV], Italy). The heavy band was set between 5 Hz and 5 KHz; 5 resting denervation potentials were measured (sensibility 50 μV/division) in the right and left gastrocnemius muscles.

### Skeletal muscles dissection

Gastrocnemius caput medialis (GCM) muscles were collected from 129SvG93A, and C57G93A mice and relative NTG littermates were euthanised respectively at 12, 14, and 16 weeks of age and 12, 18, and 22 weeks of age, corresponding to the presymptomatic (PS), symptom onset (OS), and symptomatic (SY) stages based on the performance on grip strength test. GCM muscles from each mouse were dissected and snap-frozen in isopentane cooled in liquid nitrogen and stored at −80 °C until use. After being weighed, muscles were used for immunohistochemical, biochemical, and biomolecular analyses.

### Immunohistochemistry

GCM sections were collected on poly-lysine objective slides (VWR International). For our purpose, 20-μM longitudinal serial cryosections or 12-μM coronal serial cryosections were analysed. To evaluate NMJ structure and muscle denervation, longitudinal cryosections were fixed in acetone for 10′, air-dried, and washed. After blocking with 10% NGS in PBS for 1 h, muscle slides were incubated with the primary antibodies: mouse anti-synaptic vesicle glycoprotein 2A (SV2, 1:50; SV2 DSHB), mouse anti-neurofilament medium polypeptide 2H3 (1:100; DSHB AB_2314897), and following the respective secondary antibody: anti-mouse 488 (1:500; Invitrogen) and anti-rabbit 647 (1:500; Invitrogen). Then, *α*-bungarotoxin (*α*-BTX) coupled to Alexa Fluor™ 594 (1:500; Invitrogen B13423) was incubated for 1 h at room temperature. Images were obtained with Nikon A1 confocal scan unit (Nikon Corporation, Japan) at 20× magnification. The co-localisation channel between neurofilament (SV2/2H3) and *α*-BTX immunostaining was produced for each *Z*-stack. The percentage of innervated NMJs was quantified considering the overlap between neurofilaments (SV2/2H3) staining and *α*-BTX-labelled endplates. NMJ morphology was evaluated on images obtained with Nikon A1 confocal scan unit at 40× magnification using the “NMJ-morph” workflow in Fiji (ImageJ, US National Institute of Health, Bethesda, Maryland, USA) as previously described [27].

To evaluate macrophage density, longitudinal muscle slides were incubated overnight with the following: anti-CD11b, mouse (1:200; BioRad), and anti-iNOS, rabbit (1:200; Invitrogen). Secondary antibodies were as follows: Alexa Fluor 488 anti-rat and Alexa Fluor 647 anti-rabbit (1:500; Thermo Fisher); nuclei have been marked with Hoechst 33342 (1:1000; Invitrogen 33258). A stereological random sampling procedure was applied as previously described [25]. Briefly, a grid of rectangular sampling fields was delineated on the profile of the muscle slice using the “Grid” function in ImageJ (US National Institutes of Health). To ensure that each part of the tissue slice had the same probability of being sampled, the analysis was done on defined Z-stacked image fields, acquired with A1 Nikon confocal running NIS-Elements (Nikon) at 20× magnification, at a fixed distance between them.

To evaluate the cross-sectional area (CSA), transverse muscle GCM sections were fixed in acetone for 10′, air-dried, and washed. To retrieve antigens, slides were immersed in citrate buffer (1×; Dako S2369) at 80 °C for 15′ and then at RT for other 15′ and washed. After blocking with 10% NGS in PBS for 1 h, muscle slides were incubated with Goat anti-mouse FAB fragment (1:100; Jackson Immuno 115-007-003) for 45′ and then washed.

Serial transverse GCM sections were stained with lectin (1:100; Invitrogen) for cellular membranes, and nuclei were marked with Hoechst 33342 (1:1000; Invitrogen 33258). To determine the fibre type, sections have been incubated with primary antibodies: MyHC type 1 (1:10; DSHB BA-D5), MyHC type 2a (1:17; DSHB SC-71), MyHC type 2b (1:9; DSHB BF-F3), rabbit anti-laminin (1:100; Sigma L9393), and the respective secondary antibodies: anti-MIgG2b Alexa Fluor 564 (1:500; Invitrogen A21144), anti-MIgG1 Alexa Fluor 488 (1:500; Invitrogen A21121), anti-MIgM Alexa Flour 647 (1:500; Invitrogen A21046), and anti-rabbit Alexa Fluor 405 (1:500; Abcam ab175649). To determine embryonic muscle fibres, anti-MyH3 (1:10; DSHB F1652) was used. Images were acquired with a sequential scanning mode by an A1 Nikon confocal running NIS-Elements at 20× magnification, and muscle sections were analysed with the “MuscleJ” plug-in of Fiji software as previously described [28].

### Real-time PCR

RNA from GCM was extracted using TRIzol (Invitrogen) and purified with PureLink RNA columns (Life Technologies). RNA samples were treated with DNase I, and reverse transcription was performed with High-Capacity cDNA Reverse Transcription Kit (Life Technologies). Real-time PCR was performed using the TaqMan Gene expression assay (Applied Biosystems) following the manufacturer’s instructions, on cDNA specimens in triplicate, using 1× Universal PCR Master Mix (Life Technologies) and 1× mix containing specific receptors probes (Life Technologies). Relative quantification was calculated from the ratio between the cycle number (Ct) at which the signal crossed a threshold set within the logarithmic phase of the given gene and that of the reference *β*-actin gene (4310881E; Life Technologies). Mean values of the triplicate results for each animal were used as individual data for 2-*Δ*Ct statistical analysis. The following is the list of probes used: nicotinic cholinergic receptor, gamma subunit (AChRγ) (CHRNG; Mm00437419_m1; Life Technologies), epsilon subunit (AChRε) (CHRNE; Mm00437411_m1; Life Technologies), alpha subunit (AChRα1) (CHRNA1; Mm00431629_m1; Life Technologies), beta subunit (AChRβ1) (CHRNB1; Mm00680412_m1; Life Technologies), delta subunit (AChRδ) (CHRND; Mm00445545_m1; Life Technologies), tumour necrosis factor alpha (TNF-alpha) (Mm00443258_m1; Life Technologies), insulin-like growth factor 1 (IGF1) (Mm00439560_m1; Life Technologies), CD4 (Mm00442754_m1; Life Technologies), forkhead box P3 (FOXP3) (Mm00475162_m1; Life Technologies), amphiregulin (Mm00437583_m1; Life Technologies), CD11c (Mm00498701_m1; Life Technologies), interferon-γ (IFNγ) (Mm01168134_m1; Life Technologies), transforming growth factor-β1 (Mm01178820_m1; Life Technologies), CD8a (Mm01182107_g1; Life Technologies), MyoD1 (Mm00440387_m1; Life Technologies), MyoG (Mm00446194_m1; Life Technologies), and Tmem8c (Mm00481256_m1; Life Technologies).

### Western blotting

Mice were sacrificed according to institutional ethical procedures by decapitation, and the GCM was frozen on cooled isopentane and stored at −80 °C until use. Equal amounts of total protein homogenates were loaded on a polyacrylamide gel and electroblotted onto a PVDF membrane (Millipore) as previously described [25]. Membranes were immunoblotted with the primary antibodies, followed by HRP-conjugated secondary antibodies (Santa Cruz) and developed by Luminata Forte Western Chemiluminescent HRP Substrate (Millipore) on the Chemi-Doc XRS system (Bio-Rad). Immunoreactivity was normalised to the total amount of protein loaded (Ponceau). The following antibodies were used: goat anti-Agrin (1:200; R&D Systems), rabbit anti-Rapsyn (1:1000; Abcam), mouse anti-Pax7 (1:1000; DSHB), rabbit anti-MyoD (1:1000; Proteintech), mouse anti-MyoG (1:130; DSHB), mouse anti-Sirtuin1 (1:750; Sigma Aldrich), rabbit anti-hSOD1 (1:1000; StressMarq Biosciences), rabbit anti-ARG1 (1:500; Abcam), anti-AMPK (1:1000; Cell Signaling), and anti-pAMPK (1:1000; Cell Signaling).

Immunoblot of human muscle biopsies was performed using the same protocol described above using the following primary antibody: rat anti-CD68 (1:500, Bio-Rad), rabbit anti-iNOS (1:2000, Abcam), rat anti-CD206 (1:500, Bio-Rad), and rabbit anti-Iba1 (1:500; Wako).

### Membrane preparation and injection in Xenopus oocytes and electrophysiology

The technique of “membranes microtrasplantation” in *Xenopus oocytes* permits using minute amounts of tissues bypassing the biosynthetic machinery of host cells and allowing the incorporation of native receptors and associated signaling that maintain their functional properties [29].

GCM muscles from 129SvG93A mice of 12 and 14 weeks of age and C57G93A mice of 12 and 18 weeks and relative NTG littermates were snap-frozen in liquid nitrogen immediately after collection and then shipped in dry ice to “Sapienza” University of Rome.

The membrane homogenates were prepared and injected into *Xenopus* oocytes as previously described [30]. We pooled two samples of gastrocnemius muscles for each membrane preparation from two different animals.

Acetylcholine-evoked currents (*I*_ACh_) were recorded using the two-electrode voltage clamp 24 to 48 h after the injection, using two microelectrodes filled with 3 M KCl. After being placed in the recording chamber (0.1 mL volume), the oocytes were perfused continuously (9–10 mL/min) with oocyte Ringer’s solution (OR — composition in mM: 82.5 NaCl; 2.5 KCl; 2.5 CaCl_2_; 1 MgCl_2_; 5 HEPES, adjusted to pH 7.4 with NaOH). The experiments were performed at room temperature (20–22 °C) and, unless otherwise specified, with a holding potential (*V*_H_) of −60 mV [31].

The *I*_ACh_ decay time was determined by stimulating the cells with long ACh applications (60 s, 500 μM) and indicated as the time taken for the current to decay from its peak to half-peak value (*T*_0.5_).

Decreases in *I*_ACh_ during repetitive ACh applications (500 μM for 7 s at 60-s intervals [32];) were quantified by expressing the peak amplitude of the sixth response as a percent of the peak amplitude of the first response. In some experiments, oocytes were allowed to recover from the repetitive ACh applications with a prolonged washout of 5, 10, or 15 min before an additional single pulse of ACh to evaluate their ability to recover from desensitisation [33]. The recovery at each time point was calculated as the amplitude of the post-washout *I*_ACh_ expressed as a percentage of the first current evoked from the same cell.

The ACh concentrations producing a half-maximal effect (*EC*_50_) were estimated by fitting the data to Hill equations (nH, Hill number) using least-square routines as previously described [31, 32].

The use of female *Xenopus laevis* frogs was authorised by the Italian Ministry of Health (authorisation no. 427/2020-PR).

### Primary satellite cell cultures and immunofluorescence

Satellite cell isolation and labelling were performed as described in Fabbrizio et al. [25]. Briefly, hindlimb muscles were isolated from sacrificed mice at 3 weeks and digested for 45 min at 37 °C under agitation in phosphate-buffered saline (PBS) (Sigma-Aldrich) supplemented with Dispase II (2.4 U/mL, Roche), Collagenase A (2 mg/ml, Roche), 0.4-mM CaCl2, 5-mM MgCl2, and deoxyribonuclease I (DNase I) (10 μg/mL, Roche). Cell suspensions were resuspended in HBSS and filtered with 100-μm and 40-μm filters. Single-cell suspension was stained with CD45/CD31/Ter119 phycoerythrin (PE) for lineage exclusion, Sca1 (Stem cell antigen 1)-fluorescein isothiocyanate (FITC) and α7 integrin allophycocyanin (APC). Cells were sorted using Moflo Astrios (Beckman Coulter). SCs were seeded on Matrigel-coated plates (Corning) at low-density (3500 cells/cm^2^) and cultured in Cyto-Grow (Resnova) complete medium as a growth medium (GM) for 4 days. For myogenic differentiation, after reaching the confluence, SCs have been shifted in DMEM + 2% horse serum up to 48 h. SC proliferation was evaluated for each well on stereological image fields acquired with an Olympus virtual slide system VS110 (Olympus) by counting the number of DAPI+/Ki67+ (Anti-Ki67 Ab; Abcam) cells per field. SC differentiation was assessed by evaluating fibre dimension and the fusion index (%) given by the number of nuclei per myotubes stained with anti-MyHC (DSHB).

### Statistics

Quantitative data are expressed as the mean ± SEM. Depending on the experiments, the parametric two-tailed Student’s *t*-test or two-way ANOVA followed by uncorrected Fisher’s LSD multiple comparisons or RM-ANOVA with a within and between comparisons were used for statistical analysis, with *p*-value ≤ 0.05 as the significance limit. GraphPad Prism v.9.01 (GraphPad Software) was used for the statistical analysis. The *Xenopus* oocytes electrophysiology data were examined through parametric (Student’s *t*-test) or nonparametric (Wilcoxon signed rank test, Mann-Whitney rank-sum test) tests based on the normal distribution assessed with the Shapiro-Wilk test. Statistical data analysis was performed with SigmaPlot 15 software (Inpixon), and differences between the two data sets were considered significant when *p* < 0.05. The (*n*) indicates the number of oocytes used in each experiment.

## Results

### Skeletal muscle denervation atrophy in fast- and slow-progressing SOD1G93A mice

**Fig. 1:**
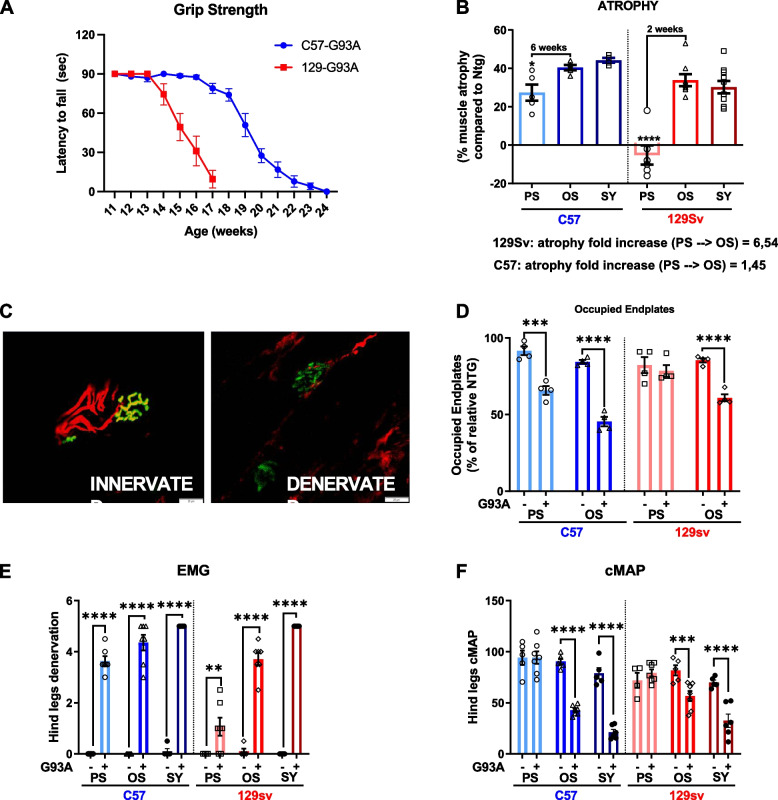
Skeletal muscle denervation atrophy in fast- and slow-progressing SOD1^G93A^ mice. **A** Paw grip endurance (PaGE) test for 129SvSOD1^G93A^ mice and C57SOD1^G93A^ mice. The data are reported as mean ± SEM for each time point (*n* = 10 mice per group). **B** Muscle mass was calculated by measure of gastrocnemius (GCM) muscles weight of 129SvSOD1^G93A^ mice; C57SOD1^G93A^ mice and NTG littermates at different time points of the disease (*n* = 4–10). Percent muscle atrophy was calculated relative to NTG mice at PS and OS. Data are reported as mean ± SEM. Significance was calculated with 2-way ANOVA with uncorrected Fisher’s LSD post-analysis (*p ≤ 0.05, ****p ≤ 0.0001). **C** and **D** Representative confocal micrographs and analysis of innervated endplates. α-Bungarotoxin (BTX, green) was used to identify the postsynaptic domain; SV2/2H3 (red) were used to identify presynaptic terminals (scale bar: 50μm). **D** Percentage of innervated endplates. Data are reported as mean ± SEM (~no. 100 BTX-positive endplates per animal were randomly chosen and analysed) (*n* = 4). Significance was calculated with 2-way ANOVA with uncorrected Fisher’s LSD post-analysis (***p ≤ 0.001, ****p ≤ 0.0001). **E** and **F** Electromyography on hindlimb muscles of C57SOD1^G93A^ mice and 129SvSOD1^G93A^ mice and NTG littermates at PS, OS, and SY. Denervation (**E**) and compound muscle action potential (cMAP) (**F**) through a coaxial electrode were evaluated. Data are reported as mean ± SEM. Significance was calculated with 2-way ANOVA with uncorrected Fisher’s LSD post-analysis and RM-ANOVA (*p ≤ 0.05, **p ≤ 0.01, *** ≤ 0.001)

The reduction in the muscle/body weight ratio of GCM muscles of both FP and SP mice was analysed compared to respective non-transgenic (NTG) littermates at different ages (FP mice: 12, 14, and 16 weeks; SP mice: 12, 18, 22 weeks) corresponding to the PS, OS, and SY stage, based on the performance on the grip strength test (Fig. 1A).

GCM atrophy, expressed as a percentage of muscle wasting of SOD1G93A mice compared to respective NTG littermates, showed a different trend between the ALS mouse models (Fig. 1B), although a comparable muscle amount of hSOD1 was detected (Supplementary Fig. 1 A, B). Notably, the FP mice showed a significant 30% reduction in GCM muscle mass at the OS, while no further muscle weight loss was registered 2 weeks later when the mice showed overt grip deficit (SY). On the contrary, 6 weeks before the appearance of clinical symptoms, SP mice showed a similar 30% GCM muscle weight loss compared to NTG mice, which modestly increased at the OS and SY stages (Fig. 1B).

Muscle denervation and NMJ dismantling are the two main features of ALS that precede and contribute to neurogenic muscle mass loss. Consistent with muscle atrophy, a significant reduction in the number of GCM-occupied endplates was observed in SP mice at the PS (−35%) and OS (−55%), while in FP mice, significant denervation was found only at the OS (−40%) (Fig. 1 C, D).

We next performed a longitudinal electrophysiological examination of motor unit connectivity in both SOD1G93A mouse strains and respective age-matched NTG mice.

Significant denervation was registered by electromyography in both SOD1G93A mice strains at PS, although this was far more consistent in SP than FP mice (mean+/−SEM; 3.6+/−0.4 vs 1.1+/−0.93 — *p < 0.01*) (Fig. 1E). At the OS and SY (Fig. 1E), the grade of denervation significantly increased both in SP and FP mice (*OS*: 4.4+/−0.8 and 3.7+/−0.6, respectively; SY 5+/−0 and 5/−0), with a higher slope in FP than SP mice (Wilks’ Lambda = 0.04, F (2, 9) = 106.84, *p* < 0.01; within-subject analysis: sphericity assumed, [F(2, 20) = 103.102, *p* < 0.01]; interaction time × strain *p* < 0.01 both for OS vs PS and SY vs PS).

The amplitude of the compound muscle action potential (cMAP) from the hind legs was significantly higher in C57 than in 129Sv strain (94.3+/−15.0 mA vs 76.6 +/−11.3 mA — *p < 0.05*) at 12-week age, regardless of the genotype (G93A or NTG) (Fig. 1F). However, no difference in cMAP was registered among respective transgenic and NTG mice at PS. Starting from the OS, both SP and FP mice showed a significant cMAP reduction, which lasted until SY (Fig. 1F). Consistently, cMAP amplitude significantly decreased in both transgenic mice at the OS and SY (SP mice_OS: 42.8+/−4.9 mV, FP mice_OS: 56.7+/−13.0mV; SP MICE_SY: 21.3+/−6.5 mV, FP mice_SY: 32.5+/−16.1mV), with a higher slope in SP than FP mice [Wilks’ Lambda = 0.05, F (2, 9) = 81.85, *p < 0.01*; within-subject analysis: sphericity assumed, [F(2, 20) = 92,800, *p* < 0.01]; interaction time × strain, *p* < 0.05 both for OS vs PS and SY vs PS).

### Expression of the nicotinic acetylcholine receptor in fast- and slow-progressing mice

Following muscle denervation, signals from peripheral nerves affect AChR subunits transcription at the synaptic nuclei and their translation and clustering at the NMJ. This process is pivotal to forming a contractile apparatus [34].

AChRs in adult-innervated muscle fibres are composed of α1β1εδ subunits densely clustered at the endplate of the NMJ to form a channel of higher conductance and faster open-time kinetics [35]. Following muscle denervation, the transcription of nAChR subunits markedly increases, leading to brand-new AChRs in which the epsilon (ε) is mostly replaced by the gamma (γ) fetal subunit [35]. These receptors are distributed over the entire sarcolemma (extrasynaptic nuclei) [36] and exhibit a low single-channel conductance and long mean channel open time [37].

**Fig. 2:**
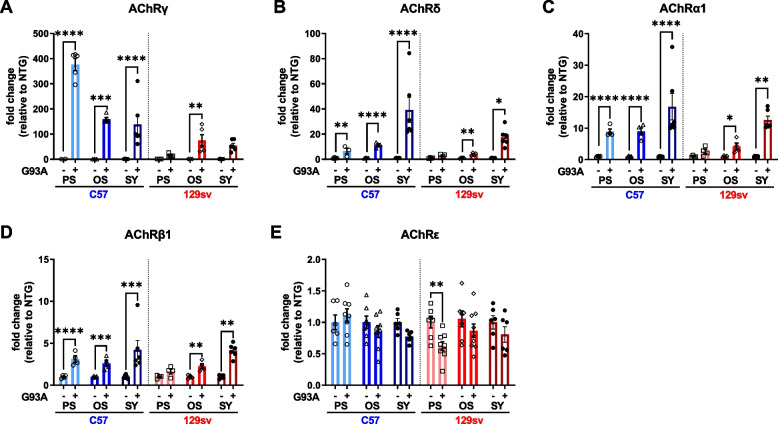
Expression of the nicotinic acetylcholine receptor in skeletal muscles of fast and slow progressing mice. **A**–**E** Real-time qPCR analysis of AchRγ (**A**), AchRδ (**B**), AchRα1 (**C**), AchRβ (**D**), AchRε (**E**) mRNA transcripts in the GCM of C57SOD1^G93A^ and 129SvSOD1^G93A^ mice compared with NTG littermates (*n* = 2–9). Data are reported as mean ± SEM. Significance was calculated with 2-way ANOVA with uncorrected Fisher’s LSD post-analysis (*p ≤ 0.05, **p ≤ 0.01, ****p ≤ 0.0001)

AChRγ mRNA levels markedly increased in the GCM of mSOD1 mice compared to NTG but to a different extent between the FP and SP mice. In SP mice, AChRγ mRNA levels increased more than 400-fold at the PS and ~150-fold at the OS. In FP mice, the *γ*-subunit significantly increased by ~100-fold compared to NTG littermates only at the OS (Fig. 2A). Noteworthy, AChRγ mRNA levels were fourfold higher in SP mice than FP mice at 12 and 14 weeks of age, respectively, notwithstanding a similar GCM denervation. Consistently, AChR α1, β, and δ subunits but not AChRε markedly increased at the PS and OS in SP mice (Fig. 2 B–E). In contrast, FP mice showed only a mild but significant increase of AChR α1, β, and δ subunits at the OS, while the AChRε transcription levels were slightly decreased at the PS, compared to respective NTG (Fig. 2 B–E). At the SY, an increase in AChR α1, β, and δ subunit mRNA levels was registered in both transgenic mice compared to the OS (Fig. 2 B–E), which is consistent with higher muscle denervation (Fig. 1E). However, no further increase in AChRγ transcript levels was found at this stage, while the levels of AChRε subunits decreased in SP mice (Fig. 2 A and E).

### Nicotinic acetylcholine receptor clustering, morphology, and activity in fast- and slow-progressing mice

One of the leading signaling cascades underlying AChR clustering in developing muscle fibres or following muscle denervation is the Agrin/lipoprotein receptor-related protein 4 (Lrp4)/Muscle-specific tyrosine kinase receptor (MuSK)/downstream of tyrosine kinases-7 (Dok-7) pathway (for review, see [34, 35]). Agrin activates MuSK phosphorylation through the transmembrane protein Lrp4. Dok-7 is an adapter necessary for the full activation of MuSK. MuSK then recruits Rapsyn, an intracellular scaffolding protein that strongly interacts with nAChRs, leading to their recruitment and clustering at the NMJ.

**Fig. 3:**
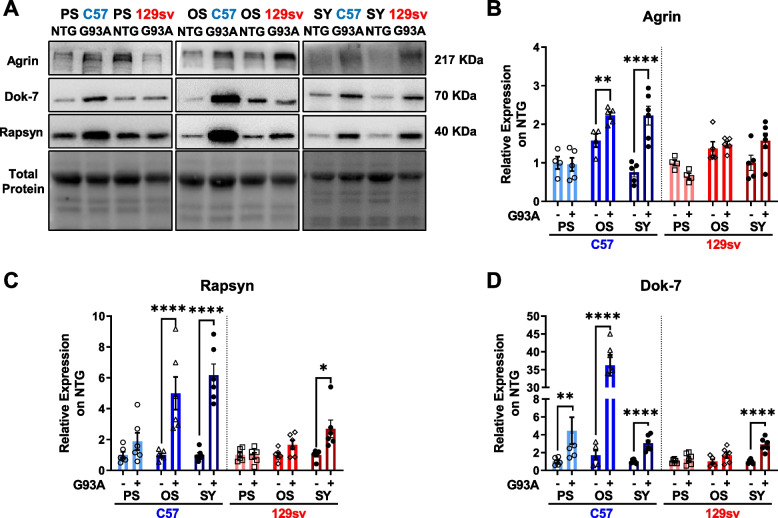
Nicotinic acetylcholine receptor clustering in the skeletal muscles of fast- and slow-progressing mice. **A**–**D** Representative immunoblot images (full blots images in Additional file 2) and relative densitometric analysis of Agrin (**A** and **B**), Rapsyn (**A** and **C**), and Dok-7 (**A** and **D**) protein expression in GCM muscles of C57SOD1^G93A^ and 129SvSOD1^G93A^ mice compared with NTG littermates (*n* = 4–6). Protein levels were normalised on the total amount of protein loaded. Data are reported as mean ±SEM. Significance was calculated with 2-way ANOVA with uncorrected Fisher's LSD post-analysis (**p ≤ 0.01, ****p ≤ 0.0001)

Based on this information, we next examined the extent of activation of this pathway in the GCM of both transgenic mice across the disease progression by analysing MuSK-related upstream and downstream signaling. In SP mice, Agrin protein levels were unchanged at the PS while significantly increased at the OS and SY (Fig. 3 A, B). In FP mice, Agrin tended to be lessened at the PS and returned to the NTG level at the OS and SY (Fig. 3 A, B). Rapsyn protein levels progressively increased from the PS until the SY in SP mice, while it showed a slight increase only at the SY in FP mice (Fig. 3 A, C). In keeping with this, a significant and marked increase in Dok-7 was registered at PS and OS only in SP mice, while both mouse models show a significant albeit modest increase at SY (Fig. 3 A, D). These data suggest an enhanced AChR clustering in the muscle fibres of SP mice across the disease progression without a variation in the NMJ morphology. On the contrary, this process is delayed or blunted in FP mice, and the motor endplate area and volume were reduced at the PS stage compared to NTG littermates (Supplementary Fig. 2 A–F).

To investigate whether the differential AChR subunits transcription and their clustering translated in a differential response to ACh between mSOD1 mouse models, we measured the ACh-elicited current applied in *Xenopus* oocytes microtransplanted with the cell membranes [30] isolated from the GCM of FP and SP mice at the PS.

**Fig. 4:**
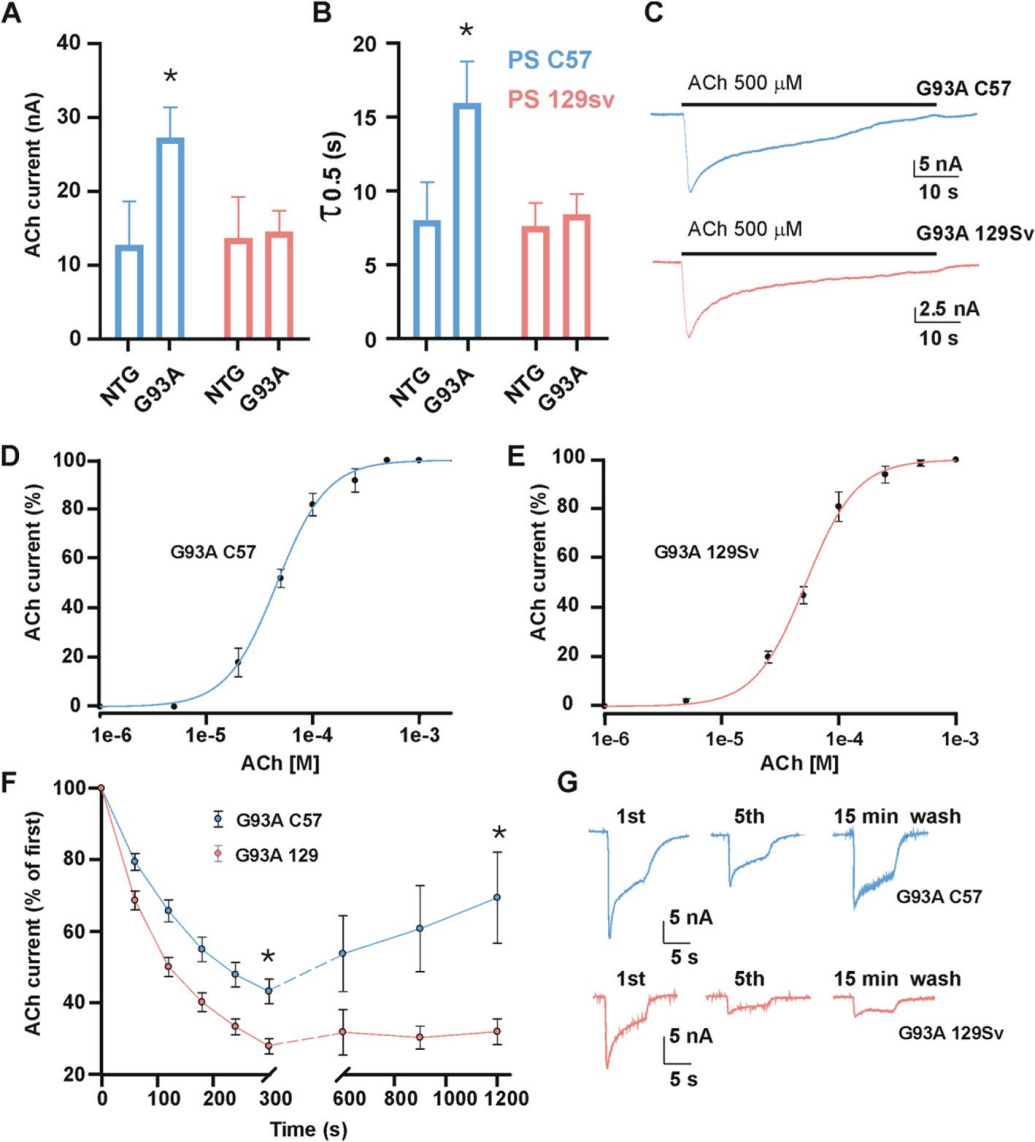
Nicotinic acetylcholine receptor activity in fast- and slow-progressing mice. **A** and **B** The bar graphs represent mean ± SEM of ACh-evoked currents amplitude (**A**) and ACh decay time (**B**). **C** Representative current traces elicited during prolonged ACh application (500 μM for 60 s) in one oocyte (representative of 12 and 16 for G93A C57 and G93A 129sv, respectively) injected with G93A C57 (blue) or G93A 129Sv muscle membranes. **D** and **E** Dose-response curves obtained by plotting the amplitude of currents evoked by ACh concentrations ranging from 1 μM to 1 mM, expressed as a percentage of the maximal current evoked at 1 mM ACh, represented as mean ± SEM and best fitted with Hill curves. Averaged *EC*_50_ values and *n*_H_ in oocytes injected with G93A C57 were 47.0 ± 0.23 μM and 1.9 ± 0.2; in oocytes injected with G93A 129Sv, the values were 54.0 ± 3.0 μM and 1.3 ± 0.1. **F** ACh-evoked current desensitisation (first six dots) followed by evaluation of current recovery after 5, 10, and 15 minutes of washout from the last pulse of the desensitisation protocol. Currents were normalised at the amplitude of the first ACh application (● = 26.1 ± 6.6 nA, *n* = 19; ● = 13.0 ± 1.2 nA, *n* = 29. (**G**) Representative ACh-evoked current traces at different time points, as indicated in the panel

SP mice-derived AChRs yielded larger ACh-evoked currents (*I*_ACh_) than their FP counterpart (27.14 ± 4.14 nA, *n* = 20 vs 14.37 ± 2.9 nA, *n* = 23, *p* = 0.020) and twofold more than age-matched NTG. Conversely, no difference was registered between FP mice and respective age-matched NTG (Fig. 4A). Besides, SP mice-derived clusters were characterised by slower *I*_ACh_ decay times when compared to FP mice (17.61 ± 3.00 s; *n* = 12 s vs 8.80 ± 1.37 s, *n* = 16, *p* = 0.025) (Fig. 4 B, C). No differences in current amplitude (12.73 ± 5.9 nA, *n* = 14 vs 13.5 ± 5.6 nA, *n* = 14, *p* > 0.05) or current decay (7.97 ± 2.6 s, *n* = 10 vs 7. 6 ± 1.6 s, *n* = 12 s, *p* > 0.05) were observed between C57 and 129Sv NTG mice (Fig. 4 A, B). The differences between transgenic animals were not related to a change of ACh receptor sensitivity since no significant variation in *EC*_50_ was registered between experimental groups (47 ± 0.23 μM, *nH* = 1.9 ± 0.2; *n* = 8; C57 mice on the right, vs 54 ± 3 μM, *nH* = 1.3 ± 0.1, *n* = 8; 129Sv mice on the left; *p* > 0.05) (Fig. 4 D, E).

Since altered receptor desensitisation can be detrimental to its function, we analysed the *I*_ACh_ amplitude decrease during repetitive ACh stimulation (i.e. *I*_ACh_ rundown [32];) at a high dose (500 μM) in oocytes transplanted with membranes from PS mice. We found that at the 6th ACh application, *I*_ACh_ amplitude decrease was significantly lower in SP than FP mice-derived AChRs (residual current after repetitive stimulation: 43.3 ± 3.4%, *n* = 19; vs 26.6 ± 2%, *n* = 29, *p* = 0.003; (Fig. 4F). Besides, SP mice-derived AChRs, but not FP ones, showed a progressive recovery of *I*_ACh_ over time (up to 15 min) (Fig. 4 F, G), suggesting that the rate of desensitisation and recovery kinetics influenced the progression of the disease.

Notably, we did not find any difference between SP and FP mice-derived AChRs in the *I*_ACh_ rundown at OS (24.7 ± 5.2%, *n* = 10; vs 25.5 ± 4.7%, *n* = 13, *p* > 0.05), and, upon 15 min of washout, both SP and FP did not show *I*_ACh_ over time. In line with this, the ACh-evoked currents between SP and FP were very similar at OS (7.4 ± 0.8 nA, range 2.1–9.5 nA, *n* = 12 vs 7.9 ± 1.3 nA, range 2.8–10.4 nA; *n* = 15; *p* > 0.05).

### Early muscle regeneration differentiates fast from slow-progressing mice

After damage, the neuromuscular system attempts to restore its function by nerve sprouting and regeneration of muscle fibres. We recently demonstrated that the extent of activation of myogenesis in the skeletal muscles impacts the disease progression in transgenic ALS mice [20, 27]. Accordingly, we next evaluated the activity of satellite cells (SCs), the *bona fide* muscle stem cells, in the GCM of SP and FP mice across the disease progression.

Under basal conditions, SCs are quiescent, becoming activated in response to acute injury or muscle denervation. Once activated, SCs proliferate, differentiate into myoblasts and fuse with existing myofibres resulting in muscle fibre repair and growth [38].

**Fig. 5:**
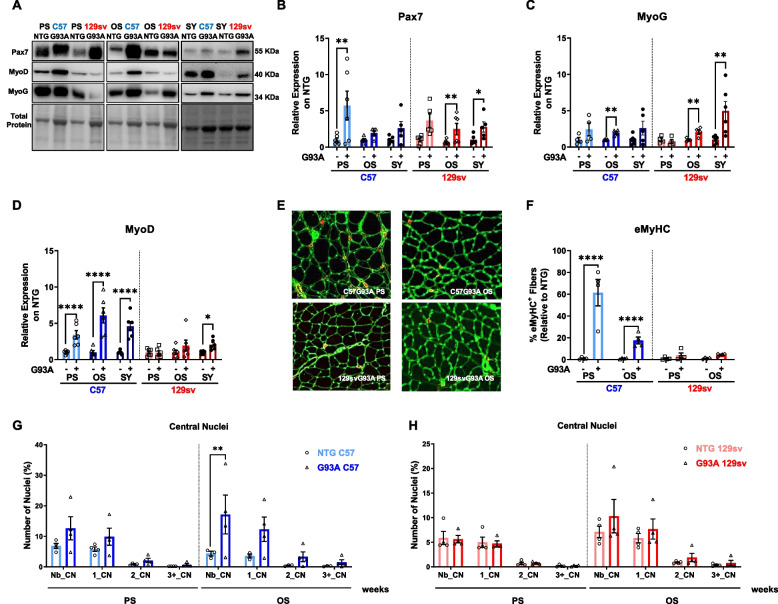
Early muscle regeneration differentiates fast from slow-progressing mice (**A**-**D**). Representative immunoblot images (full blots images in Additional file 2) and relative densitometric analysis of Pax7 (**A** and **B**), MyoG (**A** and **C**), and MyoD (**A** and **D**) protein expression in GCM muscles of C57SOD1^G93A^ and 129SvSOD1^G93A^ mice compared with NTG littermates (*n* = 4-6). Protein levels were normalised on the total amount of protein loaded. Data are reported as mean ± SEM. Significance was calculated with 2-way ANOVA with uncorrected Fisher’s LSD post-analysis (*p ≤ 0.05, **p ≤ 0.01, ****p ≤ 0.0001). **E** Representative confocal images of embryonal muscle fibres (eMyHC; red) on GCM coronal sections of C57SOD1^G93A^ mice 129SvSOD1^G93A^ mice at pre-symptomatic and onset disease stages, immunostained with laminin (green). **F** The percentage of embryonal muscle fibres was calculated relative to the total number of muscle fibres and in relation to respective NTG mice (*n* = 4). Data are expressed as the mean (±SEM). Significance was calculated with 2-way ANOVA with uncorrected Fisher’s LSD post-analysis (****p ≤ 0.0001). **G** and **H** The immunohistochemical analysis of central nuclei has been performed on coronal sections of GCM in C57SOD1^G93A^ (**G**) and 129SvSOD1^G93A^ (**H**) mice and NTG littermates at pre-symptomatic (PS) and onset (OS) disease stages (*n* = 4). Percentage of embryonal muscle fibres was calculated relative to the total number of muscle fibres and in relation to the respective NTG. Data are expressed as the mean (±SEM). Significance was calculated with 2-way ANOVA with uncorrected Fisher's LSD post-analysis (**p ≤ 0.01)

We first compared the expression of two critical myogenic regulatory factors in the GCM of both ALS mouse strains at different disease stages: Paired Box 7 (Pax7), the hallmark of SC stemness, and Myogenin (MyoG), a marker of early SC commitment and differentiation. Based on the data, Pax7 and MyoG muscle levels matched the trend of muscle denervation atrophy in transgenic mice, significantly increasing starting from the PS and OS in SP and FP mice, respectively (Fig. 5 A–C). Nevertheless, our analysis revealed a differential expression of the myoblast determination protein 1 (MyoD) between transgenic mice. MyoD levels markedly increased in the GCM of SP mice by about three-, six- and fivefold at PS, OS, and SY, respectively, compared to respective NTG littermates, while it was unchanged at PS and OS in FP mice, increasing only at SY, albeit to a lower extent than SP mice (Fig. 5 A, D).

MyoD is a transcription factor critical for promoting SC activation and defining their fate towards the generation of new mature myofibres. Accordingly, the early and selective MyoD activation in SP mice might be responsible for the enhanced muscle regeneration, testified by remarked higher percentage of embryonic (*eMyHC*^*+*^) (Fig. 5 F, E) and centro-nucleated (Fig. 5 G, H) skeletal muscle fibres in the GCM of SP mice than FP mice during the disease progression.

Together, these data are consistent with an overall activation of self-renewing SCs upon muscle denervation in both mouse models. However, SC differentiation is blunted in FP mice, leading to an improper maturation towards non-functional mature fibres.

**Fig. 6:**
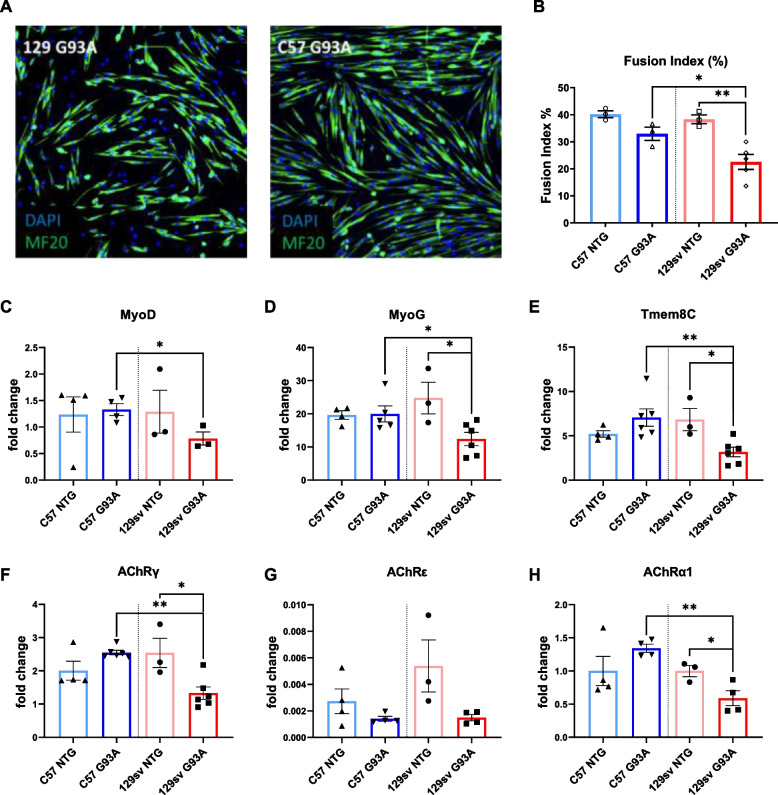
Ex vivo muscle derive satellite cells differentiate fast from slow-progressing mice. **A** Representative confocal images showing the immunostaining for MF20-MyHC (green) and DAPI (blue) on primary satellite cell (SC) cultures of C57SOD1^G93A^ and 129SvSOD1^G93A^ mice in differentiating medium (DM) for 48 h, compared with NTG littermates (*n* = 3). Scale bar = 100 μm. **B** The fusion index was calculated as (no. of nuclei present in MyHC^+^ cells with two or more nuclei/no. myotubes. Data are reported by means ± SEM. Significance was calculated with one-way ANOVA with uncorrected Fisher’s LSD post-analysis (*p ≤ 0.05, **p ≤ 0.01). **C–I** Real-time qPCR for MyoD (**C**), MyoG (**D**), Tmem8c (**E**), AchRγ (**F**), AchRε (**G**), AchRα1 (**H**) transcripts in the primary SC cultures of C57SOD1^G93A^ and 129SvSOD1^G93A^ mice compared with NTG littermates (*n* = 3–6). Data are expressed as the mean (±SEM). Significance was calculated with 2-tailed Student’s *t*-test (*p* ≤ 0.05; **p ≤ 0.01)

In keeping with this, we found that ex vivo muscle-derived SCs from FP but not SP mice had a defective differentiation in vitro (Fig. 6 A, B), as assessed by lower MyoD, MyoG, and Tmeme8C (myoblast fusion marker) protein levels than NTG littermates (Fig. 6 C–E), and this impinged on the endplate maturation (lower γ, ε, and α1 subunit expression) (Fig. 6 F–H). Noteworthy, no difference in the extent of SC proliferation was found between the two mSOD1 mice (Supplementary Fig. 3 A–E).

Defective myogenesis in FP mice was further asserted through the longitudinal morphometric analysis of GCM myofiber cross-sectional area (CSA), showing a progressive loss of large myofibres (> *1000 μm*^*2*^) starting from the PS without a compensatory increase in the percentage of myofibres with a small size (*< 250–500 μm*^*2*^) (Supplementary Fig. 4 A-D). On the contrary, in SP mice, the reduction of large myofibres was paired with a significant increase of small myofibres compared to NTG littermates at all stages examined (Supplementary Fig. 4A-D). This process may account for the generation of new mature myofibres at the OS, where no significant difference in the number of fibres with a large size was recorded between SP mice and NTG littermates (Supplementary Fig. 4C). Consistently, SP mice showed a rescue in the percentage of type 2b fast-twitch fibres (with a large diameter) compared to NTG littermates at the OS (Supplementary Fig. 4G, F). Notably, the compensatory myogenic effect was exhausted in SP mice at the SY notwithstanding an increased number of small-size myofibres, as assessed by a lessened percentage of both type 2b and large fibres (Supplementary Fig. 4H).

### Macrophage transition from M1 to M2-biased phenotype drives muscle regeneration in slow-progressing mice

Recent findings indicate that SC proliferation and differentiation committed to muscle regeneration are under the control of an inflammatory/immune response [39]. Notably, in damaged muscle, recruited immune cells must switch from a proinflammatory towards an anti-inflammatory phenotype to support the formation and growth of new myofibres [40]. We recently confirmed this paradigm in ALS mice, highlighting that an enhanced macrophage (MΦ) muscle recruitment promoted myogenesis, lessening denervation atrophy, and MN loss [20, 27].

**Fig. 7:**
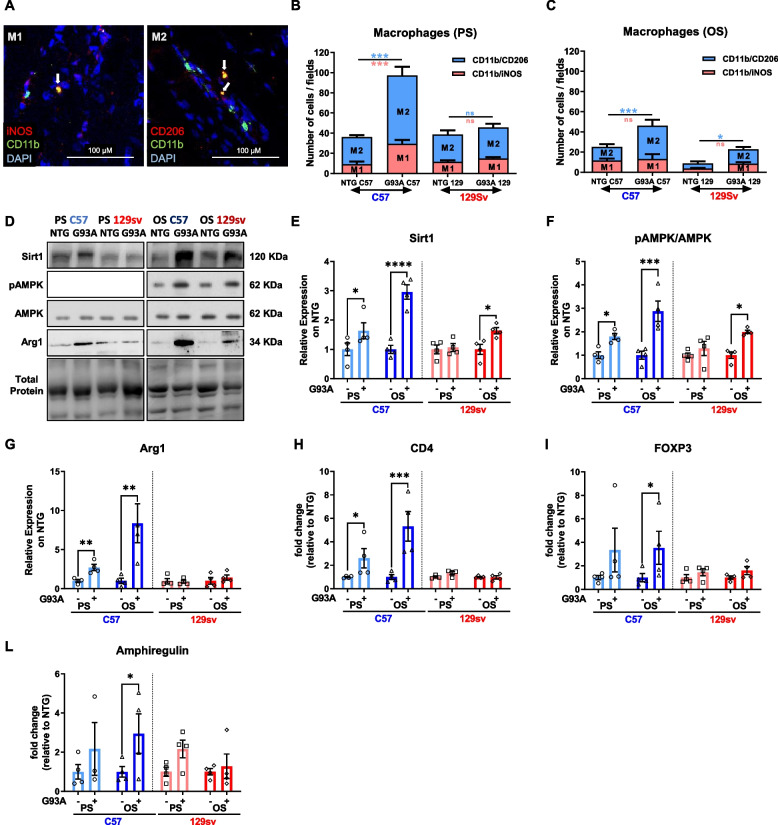
The macrophage transition from M1- to M2-biased phenotype drives myogenesis in slow-progressing mice. **A** Representative confocal micrographs showing the immunostaining for M1 (iNOS+/CD11b+/DAPI+) and M2 (CD206+/CD11b+/DAPI+) macrophages (MΦ) in longitudinal GCM sections of transgenic mice. **B** and **C** Percentage of M1 and M2 MΦ in the GCM of transgenic and NTG littermates at the presymptomatic (PS) (**B**) and onset (OS) (**C**) disease stages, calculated relative to the total number of CD11b+/DAPI+ cells counted on five stereological 0.6 × 0.6 mm fields analysed for each slice. Data are expressed as the mean ± SEM (*n* = 4). Significance was calculated with one-way ANOVA with uncorrected Fisher’s LSD post-analysis (*p ≤ 0.05, ***p ≤ 0.001). **D**–**F** Representative immunoblot images (full blots images in Additional file 2) and relative densitometric analysis of **D** and **E** Sirt-1, **D** and **F** pAMPK/AMPK, **D** and **F** Arg1, and **D** and **G** protein expression in GCM muscles of C57SOD1^G93A^ and 129SvSOD1^G93A^ mice compared with NTG littermates (*n* = 4). Data are expressed as the mean (±SEM). Significance was calculated with 2-way ANOVA with uncorrected Fisher’s LSD post-analysis (*p ≤ 0.05; ****p ≤ 0.0001). **H**–**L** Real-time qPCR for CD4 (**H**), FOXP3 (**I**), amphiregulin (**L**) mRNA transcripts in GCM muscle of C57SOD1^G93A^ and 129SvSOD1^G93A^ mice compared with NTG littermates (*n* = 4). Data are expressed as the mean (±SEM)-fold change ratio between NTG C57 mice, C57SOD1^G93A^ mice, 129SvSOD1^G93A^ mice, and NTG 129Sv mice. Significance was calculated with 1-way ANOVA with uncorrected Fisher’s LSD post-analysis (*p ≤ 0.05, **p ≤ 0.01)

Here, we found that MΦ recruitment and fingerprint markedly differ between the SP and FP mice across the disease progression within skeletal muscles. Starting from the PS, SP mice but not FP mice experienced marked MΦ muscle recruitment (Fig. 7 A–C). Notably, the MΦ M2-like phenotype (CD206^+^) was the most representative in the skeletal muscle of SP mice. Indeed, increased Sirtuin 1 (SIRT1) deacetylase protein levels, and AMP protein kinase phosphorylation (pAMPK) at the PS and OS supported the MΦ skewing from M1 towards M2-biased phenotype in the GCM of SP mice consistent with the increase of the M2-biased MΦ markers Arginase 1 (Fig. 7 D–G). Conversely, only a mild increase in SIRT1 and pAMPK but no Arginase 1 was registered in FP mice at the OS (Fig. 7 D–G). Consistently, the mRNA levels of CD4 T cell receptor (Fig. 7H), the forkhead box protein 3 (FOXP3) Treg marker (Fig. 7I) and Amphiregulin (Fig. 7L), a growth factor produced by Treg cells to support myogenic differentiation [41], significantly increased at PS and OS in SP mice, but not in FP mice, suggesting the establishment of an anti-inflammatory and pro-regenerative environment. Indeed, the Insulin-like growth factor 1 (Igf1), produced by M1-biased MΦ to trigger the M2-gene programme, was increased in SP mice at PS and was unchanged in FP mice (Supplementary Fig. 5A). In contrast, the M2-biased MΦ markers, *transforming growth factor β* (TGFβ), was significantly higher in the GCM of SP than in FP mice (Supplementary Fig. 5B). Conversely, the mRNA level of proinflammatory *Interferon-γ* (IFNγ) significantly increased in both transgenic mice at all considered time points (Supplementary Fig. 5C) consistent with the increase of dendritic cell marker CD11c (Supplementary Fig. 5D). At the same time, no significant alterations were found in the levels of CD8 T-cell receptor (Supplementary Fig. 5E). This surmises the establishment of an early and long-lasting proinflammatory state in the GCM of FP mice that, unlike SP mice, is not balanced by the induction of an anti-inflammatory and pro-regenerative environment.

**Fig. 8:**
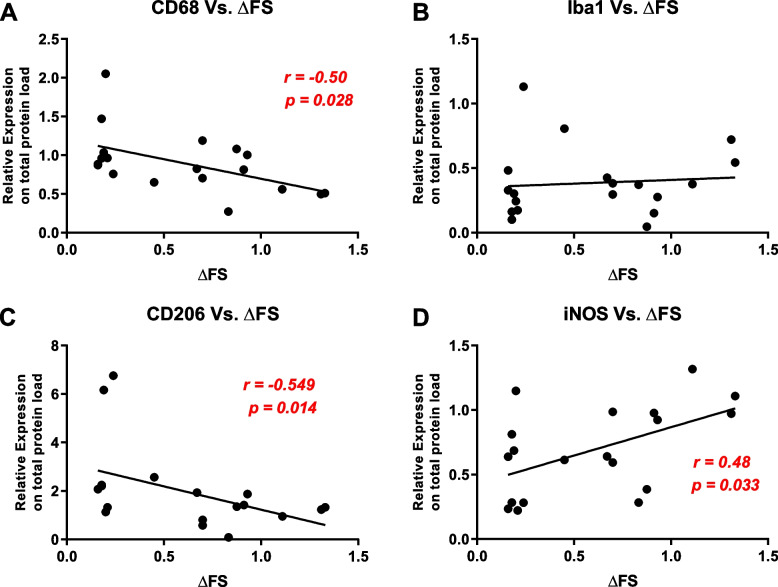
The macrophage fingerprint correlates with the slow disease progression in sporadic ALS patients. **A**–**D** Bivariate analysis showing the strength of association between the muscular protein expression of CD68 (**A**), Iba1 (**B**), CD206 (**C**), and iNOS (**D**) assessed by immunoblot and the *Δ*FRS score of ALS patients. The higher is the *Δ*FRS, the faster is the disease progression. The data were analysed by nonparametric Spearman’s rank correlation

To investigate whether these preclinical skeletal muscle alterations had translational implications for prognosis in sporadic ALS patients, we undertook an immunoblot pilot study in left *vastus lateralis* muscle biopsy of 19 age-matched ALS patients with rapid and slow disease progression identified by the Δ ALS Functional Rating Scale (ΔFS) [26]. Interestingly, we found greater infiltration of activated MΦ into the skeletal muscle of patients with slowly progressing ALS, as demonstrated by the inverse correlation between the protein level of expression of the CD68 marker and the rate of disease progression (*r*= −0.502, *p* = 0.028) (Fig. 8A), while no correlation was found in the levels of Iba1, a marker of overall macrophages (*r* = 0.172, *p* = 0.481) (Fig. 8B). In keeping with the preclinical data, the expression level of CD206, a marker of M2 MΦ, was inversely correlated with the rate of disease progression (*r* = –0.549, *p* = 0.015) (Fig. 8C). In contrast, the opposite was recorded for the M1-iNOS counterpart (*r* = 0.489, *p* = 0.034) (Fig. 8D), highlighting an M2/M1 ratio prevalent in patients with slow disease progression.

## Discussion

This study further endorses the evidence that a comparative analysis between FP and SP ALS mice carrying the same amount of mutant SOD1 is a functional experimental paradigm for identifying molecular processes and signatures governing the variability of the disease course. We previously showed that genetic background largely influences the CNS pathology and the disease course in ALS mouse models more than every single genetic modifier [8]. Here, we highlighted that immune-mediated myogenesis coupled with increased expression of fetal AChR subunits and their clustering are essential processes in skeletal muscle homeostasis, contributing to determining the difference in the age at onset and the speed of symptom progression of these two transgenic SOD1G93A mice.

In line with the dying-back hypothesis [42, 43], both fast- and slow-progressing transgenic ALS mouse models revealed NMJ defects at PS, preceding MN loss in the lumbar spinal cord [23]. However, the peripheral dysregulation was markedly different between the two ALS models, highlighting the relevance of phenotype heterogeneity in defining the extent of disease progression. SP mice had early muscle denervation atrophy, which progressed into delayed muscle dysfunction. On the contrary, similar denervation atrophy in FP mice exited to an abrupt muscle force impairment and the symptom onset. This result surmises that the engagement of compensatory response in the skeletal muscle of SP mice at the PS effectively counteracts progressive muscle denervation up to the onset of symptoms.

At the presynaptic level, SP mice preserved the cMAP amplitude and the muscle strength until the OS, notwithstanding effective muscle denervation atrophy since the PS, suggesting intense adaptive muscle reinnervation. Indeed, cMAP amplitude correlates with the reinnervation level and provides a sensitive assessment of complete NMJ recovery upon muscle denervation [44]. We impose this result on a massive increase in fetal-type AChR and ACh responsiveness in SP mice at the PS, suggesting an early establishment of fetal “ectopic” (extrajunctional) AChR clusters (α1β1γδ) [34, 45].

Fetal-type AChR clusters develop during myotubes formation or after the skeletal muscle denervation in extrajunctional regions of muscle fibres [46]. Usually refractory to innervation [34], in the presence of denervation, these regions form “ectopic” AChR clusters recognised by spared or regenerating motor axons, leading to new functional NMJs [34]. Ectopic synapses are consistent with prolonging evoked endplate current (EPC) decay after reinnervation [45] and might preserve muscle excitability and spinal MNs in SP mice at the early stage [23].

In FP mice at PS, the reduction in muscle fibre CSA matched with the reduced endplate surface and decreased expression of the AChRƐ subunit, suggesting early postsynaptic defects that precede denervation and might be predictive of the rapid worsening in disease progression. Indeed, at the OS, muscle denervation correlated with a reduced cMAP and weakened increase in fetal AChR clustering resulting in hindlimb muscle force deficiency. We attribute this difference to the lumbar spinal MNs, which are intrinsically more vulnerable in FP mice due to striking alterations in energy metabolism, protein degradation, and early microgliosis [23, 25, 47]. Indeed, FP mice showed 50% MN loss 2 weeks after initial muscle fibre wasting, while in SP a similar MN loss occurred 6 weeks after overt muscle atrophy, in correspondence of the OS [23]. Besides, FP mice showed remarkable motor axon dysregulation, myelin degradation, and defective immune cell recruitment in the periphery [20, 48], suggesting a predominant presynaptic disruption. These events may prevent spared MNs from reinnervating denervated muscle fibres leading to rapid MN loss and neuromuscular function with accelerated symptom onset and faster disease progression.

The formation of functional NMJs requires accumulating and clustering AChR subunits within the postsynaptic membranes at nerve-muscle contact sites. Notably, Agrin, a large extracellular matrix proteoglycan produced by nerve terminals, interacts with MuSK to organise the Rapsyn-mediated AChR clustering at the postsynaptic junction [34, 35]. This process is mediated by specific muscle components such as MuSK, Rapsyn, and DOK-7DOK-7, whose activity is essential for forming mature and specialised NMJs [49]. For instance, the induced overexpression of Rapsyn in the *tibialis anterior* muscles of a rat model of experimental autoimmune *myasthenia gravis* prevented AChR loss, endplate damage, and cMAP decrement [50]. Alternatively, the activation of MuSK in hindlimb muscles of C57 SOD1G93A mice by the intravenous administration of AAV encoding the human DOK-7 gene reduced muscle denervation atrophy and enhanced motor activity and life span [51].

We found a progressive and concerted rise in the expression level of Rapsyn and DOK-7 across the disease progression exclusively in SP mice. This result, which might account for maintaining ectopic AChR clusters and promoting muscle reinnervation since the early disease stages, was corroborated by the increased ACh-evoked currents and decreased receptors desensitisation in the SP mice-derived AChRs during repetitive agonist stimulation. These data suggest that residual high-frequency firing of spared MNs operates more efficiently by acting on a more “stable” NMJ in SP mice. In contrast, the faint AChR clustering response and impaired motor unit reinnervation in the GCM of FP mice led to rapid symptomatology due to a failure to form new mature endplates. Notably, the compensative ACh-evoked currents in SP mice disappeared in concomitance with the OS when the fetal-type AChRs subunits overexpression decreased, suggesting a close relationship among these events.

Preserving muscle excitability can be critical for myogenesis. In this regard, it has been reported that low-frequency electrical stimulation of rat hindlimb muscles, damaged by muscular strain, induces an increase in myogenic factors and SC activation leading to muscle regeneration [52]. In keeping with this, electrical stimulation of rat myoblast cell line increased the thickness and length of myotubes during myogenic differentiation by promoting the expression of myogenic markers (MyoD, MyoG) and activating the fusion of the myoblast cells [53]. At the same time, myogenic regulatory factors are the principal activators of the transcription of AChR subunits [54–57].

Based on the data, SP mice showed a higher SCs propensity to differentiate towards new mature muscle fibres than FP mice, which may account for the slower muscle mass reduction during the disease progression. Indeed, ex vivo muscle-derived SCs from FP mice exhibited lower expression of early (MyoD, MyoG) and late (Tmem8C, Musk) myogenic differentiation markers, and this affected the maturation of AChR clusters as assessed by the downregulation of α1, ε, and γ subunits.

Along the disease progression, variance in SC differentiation among FP and SP mice is mainly due to the differential MyoD expression in ex vivo GCM tissues, which progressively raised in the SP and was faint in FP mice despite the increase of Pax7 and MyoG. This eventually affected myogenesis and prevented the transcription and clusterisation of brand-new AChR subunits. Indeed, MyoD-deficient or mutant muscle failed to regenerate efficiently following injury due to an increased propensity for stem-cell self-renewal rather than progression through the myogenic programme [58]. These results are consistent with the observation in ALS patients’ skeletal muscle, showing a low number of PAX7^+^ cells expressing MyoD and the absence of regenerating fibres [59]. In keeping with this, ALS patient-derived SCs display abnormal senescent-like morphology in vitro due to a defective differentiation [60].

We next investigated the mechanism underlying the differential activation of the myogenic programme in SP and FP mice. We focused on the role of immune response in the skeletal muscle based on recent evidence [20, 40].

Indeed, the inflammatory response is coupled temporally and spatially to myogenesis in regenerating muscles, and it has a central role in bridging initial muscle injury responses and muscle reparation [39]. MФ are the most abundant immune cell recruited in regenerating skeletal muscle, and complete muscle tissue repair is strongly dependent on the timely recruitment of blood monocytes that enter the damaged tissue and differentiate into distinct MФ subtypes [61]. Proinflammatory MФ accumulate first in the injured tissue area to phagocytose debris and stimulate SC proliferation, subsequently converting to anti-inflammatory MФ (or pro-regenerative MФ) that support the formation and growth of new myofibres [61]. For instance, MΦ are pivotal in preserving SC identity, and MΦ depletion exacerbate the dystrophic phenotype in *mdx* mice, impairing the proliferation/differentiation balance of myogenic progenitors and causing adipogenic conversion of SCs [62]. In keeping with this, we recently demonstrated the crucial role of the immune response in promoting and governing skeletal muscle regeneration and, thus, the speed of the disease progression in mSOD1 mice [20, 27]. Mainly, we found that the in vitro P2X7 engagement by the agonist BzATP elicited the MΦ polarisation towards an M2 phenotype, and in vivo BzATP administration in skeletal muscles of SOD1G93A mice increased the M2 MΦ density, and this accounted for enhanced myogenesis and improved motor activity of mSOD1 mice [27]. Our data demonstrated that the phenotypic switch towards an M2 pro-regenerative phenotype sustained the early activation of the myogenic programme with an increased rate of muscle fibre differentiation.

The comparative analysis of mSOD1 mice revealed that SP mice experienced an early immune cell invasion, which resulted in establishing an anti-inflammatory and pro-regenerative environment able to enhance SC differentiation and muscle fibre maturation. Conversely, a faint MΦ recruitment was associated with a defective switch towards an M2 pro-regenerative phenotype in FP mice. Besides, the failure in recruiting T lymphocytes, including immunomodulatory Treg cells, translated into an imbalance of the inflammatory milieu towards a proinflammatory environment (high CD11c and IFNγ muscle levels), which eventually resulted in the development of a sterile and ineffective myogenic programme coupled with the hindered reinnervation.

Interestingly, this observation was translated in muscle biopsies from sporadic ALS patients, where we found that the levels of MΦ M2 marker, CD206, negatively correlated with the rate of disease progression. In contrast, the MΦ M1 iNOS marker raised in muscle biopsies of patients with a rapid disease progression suggesting a positive relationship between the proinflammatory status and the disease severity.

## Conclusions

The transgenic mouse models examined in this study resemble the heterogeneity in ALS patients. We previously showed that background-related factors within the CNS are decisive in defining a difference in onset and disease progression between the two animal models [8, 23, 25]. Indeed, earlier spinal cord inflammation is concomitant with a higher defective protein catabolic response, mitochondrial dysfunction, and axonal function impairment in the CNS of fast than slow-progressing mice. In addition, 129Sv mice have a higher basal metabolic rate and lower circulating insulin levels than the C57Bl6 mice [63], which may account for increased hypermetabolism, one of the hallmarks associated with ALS pathogenesis [64]. Here, we pinpointed that genetic background also affects the extent of activation of presynaptic and postsynaptic compensatory mechanisms to impinge on muscle atrophy and contribute to the variability of the disease course.

**Fig. 9:**
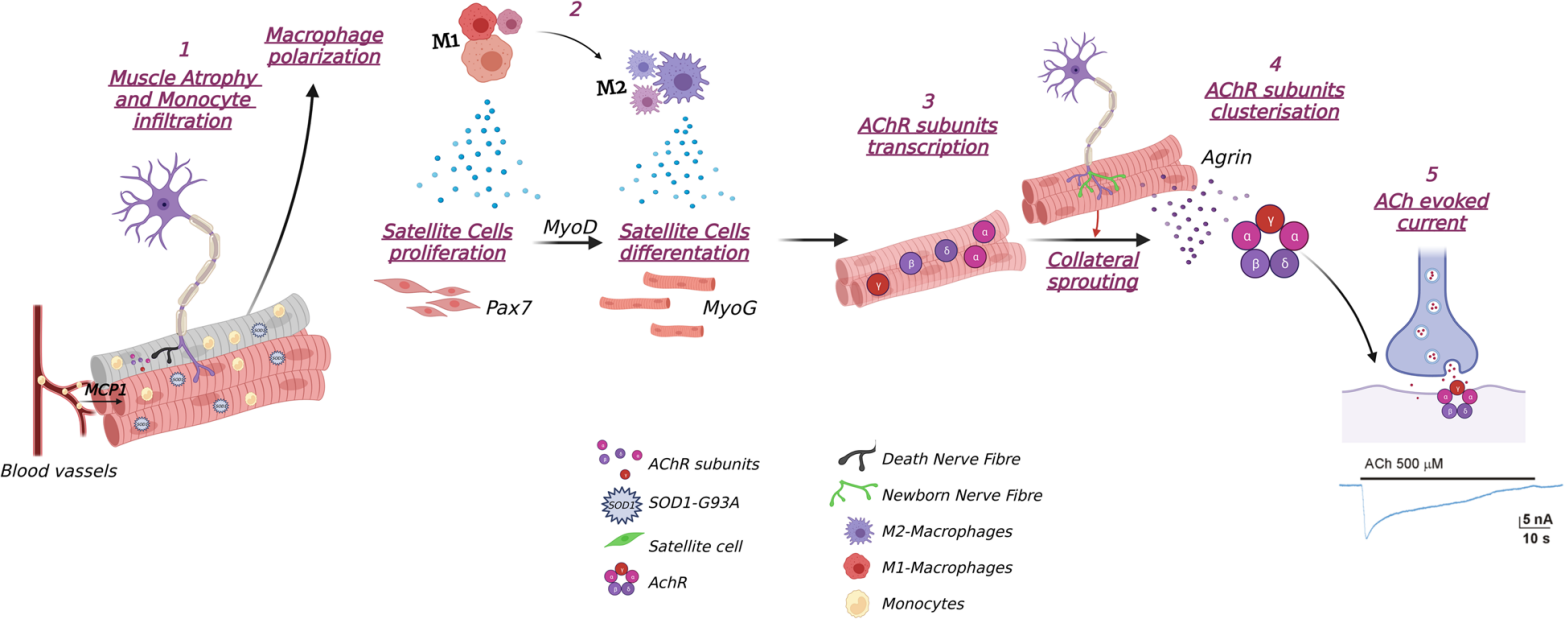
Candidate cellular mechanisms for motor unit preservation in slow-progressing ALS mice. (1) Muscle denervation induces proinflammatory MФ (M1) invasion, mediated by chemotactic signals (i.e. MCP1), in the injured tissue area to phagocytise debris and stimulate SC proliferation; (2) the conversion to anti-inflammatory MФ (M2) (activation Sirt1, AMPK, Arg1) supports the formation and growth of new myofibres; (3) myogenic regulatory factors, MyoG and MyoD, elicit AChR subunits transcription in newly formed and denervated myofibres; (4) AChR clusterisation is induced by muscle reinnervation (collateral sprouting), which occurs at both denervated and newly formed synapses; (5) the evoked response to released ACh is enhanced leading to the maintenance of motor unit function despite muscle atrophy. The lack of M1-M2 polarisation in fast-progressing ALS mice prevents myogenesis and reinnervation upon muscle denervation. *Created with *BioRender.com

Focusing on the NMJ and skeletal muscles, we found remarkable differences between two mSOD1 models endorsing the relevance of immune cell tissue infiltration in sustaining an anti-inflammatory M2 response, crucial for efficient muscle fibre regeneration, which in turn elicited synaptic ACh clusterisation and response following muscle innervation (Fig. 9).

Our data add new insights to understanding the mechanisms underlying the “incubation period” during the dying-back phenomenon, which precedes the manifestation of symptoms. We showed that SP mice, similar to congenic C57BL/6J-SOD1G93A mice [65], had a long incubation period notwithstanding early muscle atrophy. Conversely, switching the background to the 129Sv leads to a remarked shortening of the prodromal stage, the phase between the possible signs and the definite symptoms (phenoconversion) [66]. The lack of a compensatory response at the skeletal muscle associated with a rapid MN dysfunction at the CNS level determined a more aggressive clinical phenotype in 129SvSOD-1G93A mice.

Besides, we demonstrated how skeletal muscle could be a paramount source of ALS biomarkers, being one of the most severely affected tissues by the disease, easily accessible to biopsy even during the disease progression.

In a multidrug treatment perspective aimed at counteracting the multisystem nature of ALS, a well-design approach for targeting skeletal muscle could be an additional strategy for preserving the whole motor unit.

The results of the present research highlighted underestimated peripheral mechanisms and druggable targets in ALS, providing new insights for better stratification of ALS patients’ phenotype variability to facilitate novel therapeutic strategies and clinical management.

### Study approval

All procedures performed in animal studies were in accordance with the ethical standards of the Mario Negri Institute and approved by the Institutional Review Board and the Italian Ministry of Health (authorisation no. 246/2020-PR).

All human specimens were obtained for diagnostic purposes upon material transfer agreement between the Neuromuscular Bank of Tissues at the University of Padua (Telethon Network of Genetic Biobanks; TNGB) and the last author. The human material was stored at the Mario Negri biorepositories. Details on the ethical guidelines adopted by the Biobank, including confidentiality/data protection, storage, and distribution of material, are provided at http://biobanknetwork.telethon.it/Pages/View/TheCharter#ethical_guidelines.

## Supplementary Information

**Additional file 1: Supplementary Figure 1.** Skeletal muscle SOD1 expression and late muscle denervation in fast- and slow-progressing SOD1^G93A^ mice. **Supplementary Figure 2.** Nicotinic Acetylcholine receptor morphology in fast- and slow-progressing mice. **Supplementary Figure 3.** Ex-vivo muscle-derive satellite cells proliferation in fast and slow-progressing mice. **Supplementary Figure 4.** Muscle fibre composition of the two ALS murine models. **Supplementary Figure 5.** Muscle inflammatory signals in fast- and slow-progressing mice.

**Additional file 2.** Original full-length blot images for Figs. 3A, 5A, 7D and 8A-D.

## Data Availability

The datasets used and/or analysed during the current study are available from the corresponding author on reasonable request.

## Abbreviations

AChRs: Nicotinic acetylcholine receptors
ALS: Amyotrophic lateral sclerosis
CSA: Cross-sectional area
EMG: Electromyography
GCM: Gastrocnemius caput medialis
MNs: Motor neurons
MΦ: Macrophage
NMJs: Neuromuscular junctions
NTG: Non-transgenic
OS: Symptom onset
PNS: Peripheral nervous system
PS: Pre-symptomatic
SCs: Satellite cells
SOD1: Superoxide dismutase 1
SY: Symptomatic
*Δ*FS: *Δ*ALS Functional Rating Scale
