# Correction: Immune-mediated myogenesis and acetylcholine receptor clustering promote a slow disease progression in ALS mouse models

**DOI:** 10.1186/s41232-023-00276-4

**Published:** 2023-04-19

**Authors:** Cassandra Margotta, Paola Fabbrizio, Marco Ceccanti, Chiara Cambieri, Gabriele Rufolo, Jessica D’Agostino, Maria Chiara Trolese, Pierangelo Cifelli, Veronica Alfano, Christian Laurini, Silvia Scaricamazza, Alberto Ferri, Gianni Sorarù, Eleonora Palma, Maurizio Inghilleri, Caterina Bendotti, Giovanni Nardo

**Affiliations:** 1 Laboratory of Molecular Neurobiology, Department of Neuroscience, Istituto di Ricerche Farmacologiche Mario Negri IRCCS, Via Mario Negri 2, 20156 Milan, Italy; 2 Department of Human Neurosciences, Rare Neuromuscular Diseases Centre, Sapienza University of Rome, 00185 Rome, Italy; 3 Laboratory Afliated to Istituto Pasteur Italia, Department of Physiology and Pharmacology, Sapienza University of Rome, 00185 Rome, Italy; 4 IRCCS San Rafaele Roma, 00163 Rome, Italy; 5 Department of Applied Clinical and Biotechnological Sciences, University of L’Aquila, 67100 L’Aquila, Italy; 6 IRCCS Fondazione Santa Lucia, Rome, Italy; 7 Institute of Translational Pharmacology (IFT-CNR), Rome, Italy; 8 Department of Neuroscience, Azienda Ospedaliera di Padova, Via Giustiniani 2, 35128 Padua, Italy

**Correction** **:** **Inflamm Regen 43, 19 (2023)**

**https://doi.org/10.1186/s41232-023-00270-w**

Following publication of the original article [1], the authors reported that Fig. 7 needed to be amended.

**Fig. 7:**
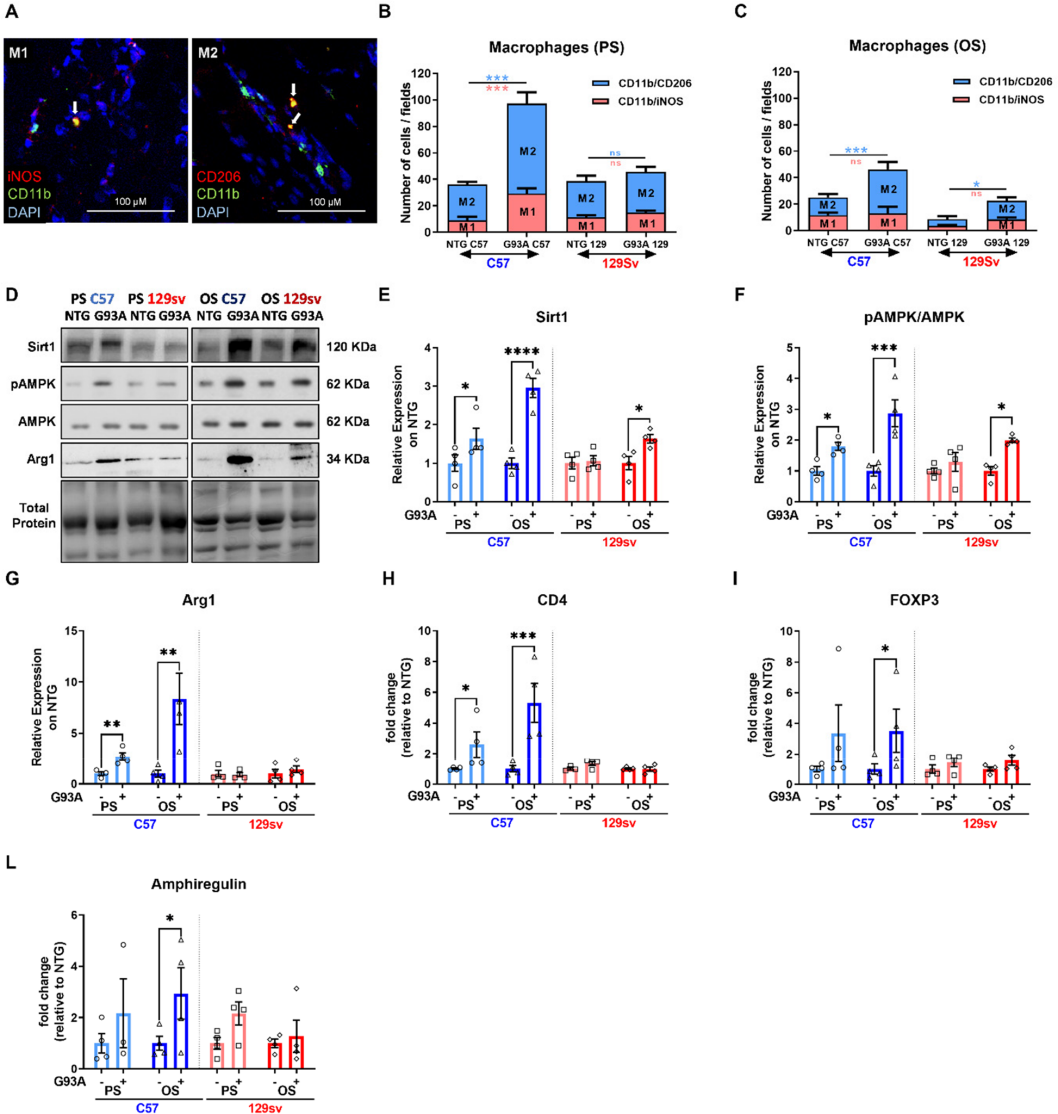
The macrophage transition from M1- to M2-biased phenotype drives myogenesis in slow-progressing mice. **A** Representative confocal micrographs showing the immunostaining for M1 (iNOS + /CD11b + /DAPI +) and M2 (CD206 + /CD11b + /DAPI +) macrophages (MΦ) in longitudinal GCM sections of transgenic mice. **B** and **C** Percentage of M1 and M2 MΦ in the GCM of transgenic and NTG littermates at the presymptomatic (PS) (**B**) and onset (OS) (**C**) disease stages, calculated relative to the total number of CD11b + /DAPI + cells counted on five stereological 0.6 × 0.6 mm fields analysed for each slice. Data are expressed as the mean ± SEM (*n* = 4). Significance was calculated with one-way ANOVA with uncorrected Fisher’s LSD post-analysis (*p ≤ 0.05, ***p ≤ 0.001). **D–F** Representative immunoblot images (full blots images in Additional file 2) and relative densitometric analysis of **D** and **E** Sirt-1, **D** and **F** pAMPK/AMPK, **D** and **G** Arg1 protein expression in GCM muscles of C57SOD1G93A and 129SvSOD1G93A mice compared with NTG littermates (*n* = 4). Data are expressed as the mean (± SEM). Significance was calculated with 2-way ANOVA with uncorrected Fisher’s LSD post-analysis (*p ≤ 0.05; ****p ≤ 0.0001). **H–L** Real-time qPCR for CD4 (**H**), FOXP3 (**I**), amphiregulin (**L**) mRNA transcripts in GCM muscle of C57SOD1G93A and 129SvSOD1G93A mice compared with NTG littermates (*n* = 4). Data are expressed as the mean (± SEM)-fold change ratio between NTG C57 mice, C57SOD1G93A mice, 129SvSOD1G93A mice, and NTG 129 Sv mice. Significance was calculated with 2-way ANOVA with uncorrected Fisher’s LSD post-analysis (*p ≤ 0.05, **p ≤ 0.01)

The correct Fig. 7 has been provided in this Correction.

The original article [1] has been corrected.
