# Supplementary material for: Immune-mediated myogenesis and acetylcholine receptor clustering promote a slow disease progression in ALS mouse models
Source: Inflamm Regen. 2023 Mar 9;43:19. doi: 10.1186/s41232-023-00270-w (PMC9996869; doi:10.1186/s41232-023-00270-w)
Supplement: Supplementary file 1 — Additional file 1: Supplementary Figure 1. Skeletal muscle SOD1 expression and late muscle denervation in fast- and slow-progressing SOD1G93A mice. Supplementary Figure 2. Nicotinic Acetylcholine receptor morphology in fast- and slow-progressing mice. Supplementary Figure 3. Ex-vivo muscle-derive satellite cells proliferation in fast and slow-progressing mice. Supplementary Figure 4. Muscle fibre composition of the two ALS murine models. Supplementary Figure 5. Muscle inflammatory signals in fast- and slow-progressing mice. [file 41232_2023_270_MOESM1_ESM.pdf]

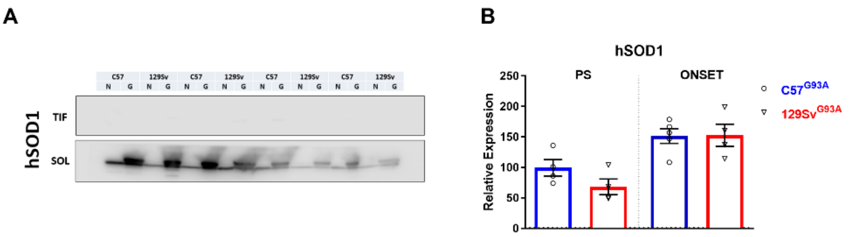

Supplementary Fig. 1. Skeletal muscle SOD1 expression and late muscle denervation in fast and slow progressing SOD1^{G93A} mice. (**A** and **B**) Representative Immunoblot image and relative densitometric analysis of soluble and aggregated human and murine SOD1 protein expression in GCM muscles of C57SOD1^{G93A} and 129SvSOD1^{G93A} mice compared with NTG littermates (*n* = 4). Protein levels were normalised on the total amount of protein loaded. Data are expressed as the mean (± SEM) and analysed by 2-way ANOVA with uncorrected Fisher's LSD post-analysis.

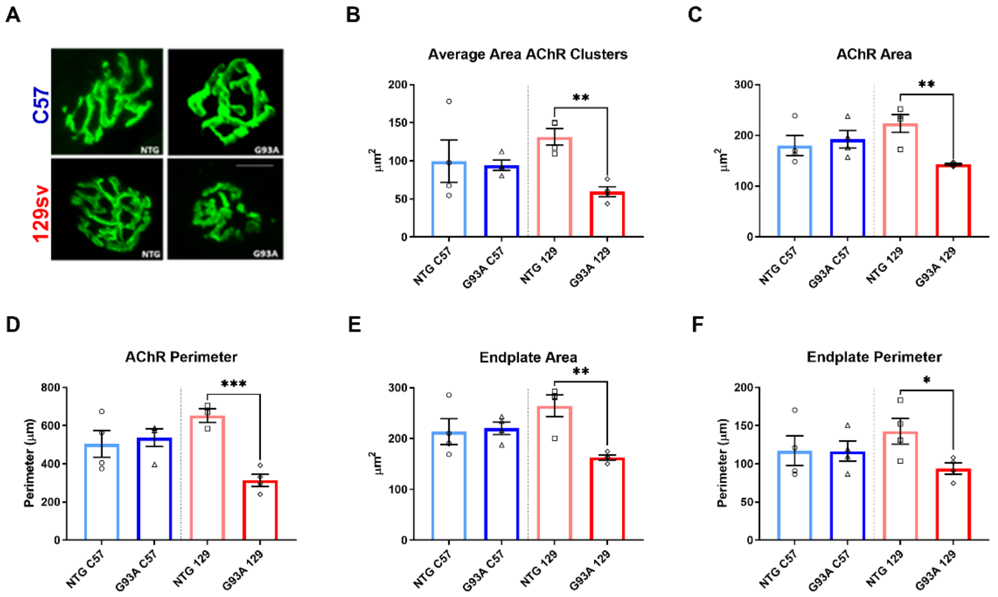

Supplementary Fig. 2. Nicotinic Acetylcholine receptor morphology in fast and slow progressing mice. (**A**) Representative confocal micrographs and morphometric analysis of α-Bungarotoxin (BTX) positive endplates on longitudinal sections of GCM in C57SOD1^{G93A} mice, 129SvSOD1^{G93A} mice and NTG littermates at 12 weeks of age. (**B**-**F**) Average area clusters (**B**)**,** AChR area (**C**), AChR perimeter (**D**), Endplate area (**E**) and Endplate perimeter (**F**) have been analysed through NMJ-Morph (*n* = 4). Data are expressed as the mean (± SEM). Significance was calculated with Two-way ANOVA with uncorrected Fisher's LSD post-analysis (**P* ≤ 0.05; ***P* ≤ 0.01; ****P* ≤ 0.001).

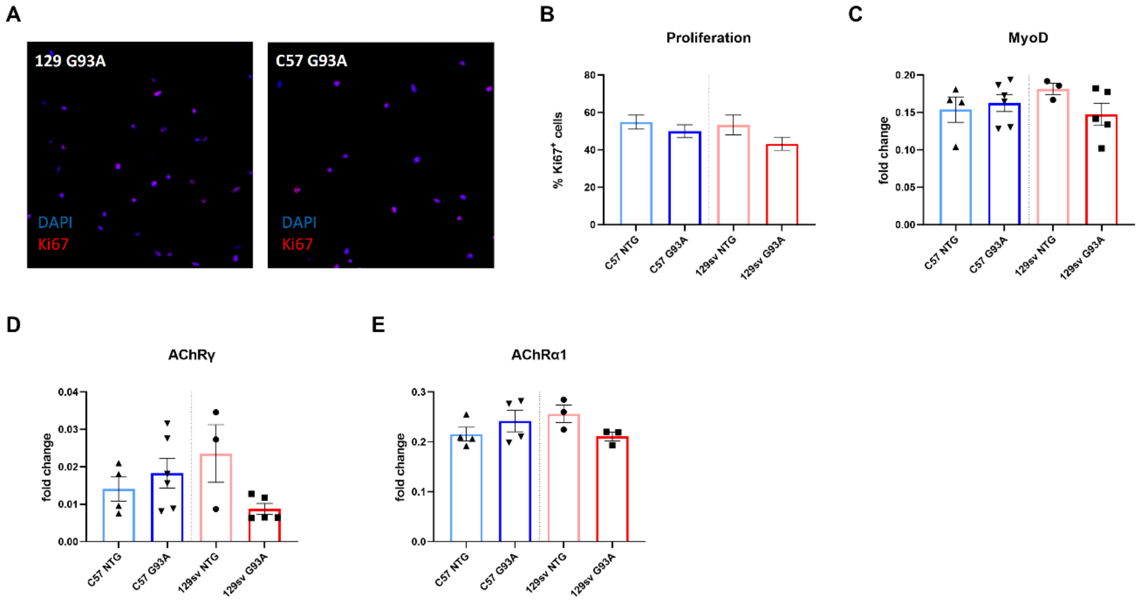

Supplementary Fig. 3. Ex-vivo muscle-derive satellite cells proliferation in fast and slow-progressing mice. (**A**) Representative confocal micrographs showing the immunostaining for Ki67 (red) and DAPI (blue) on primary satellite cell (SC) cultures of C57SOD1^{G93A} and 129SvSOD1^{G93A} mice in growing medium for 72 h, compared with NTG littermates. Scale bar = 100 µm. (**B**) The proliferation index was calculated as No. of Ki67 and DAPI positive cells (*n* = 3). Data are reported by means ± SEM. Statistics were calculated by One-way ANOVA with uncorrected Fisher's LSD post-analysis. (**C**-**E**) Real-time qPCR for MyoD (**C**), AchRγ (**D**) and AchRα1 (**E**) mRNA transcripts in primary SC cultures of C57SOD1^{G93A} and 129SvSOD1^{G93A} mice compared with NTG littermates (*n* = 3). Data are expressed as the mean (± SEM). Data were analysed by Two-way ANOVA with uncorrected Fisher's LSD post-analysis.

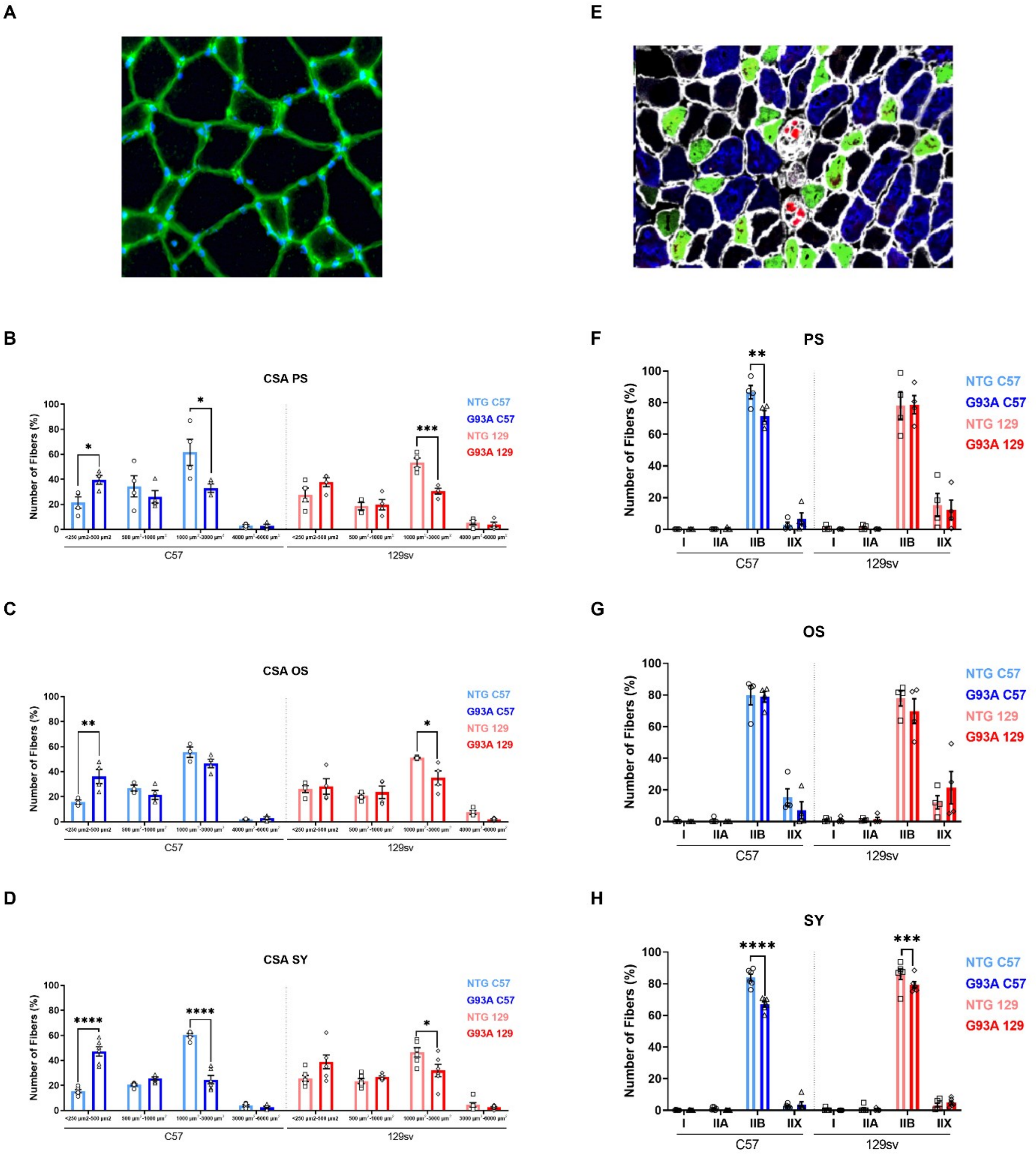

Supplementary Fig. 4. Muscle fibre composition of the two ALS murine models. (**A** and **D**) Representative confocal micrograph and longitudinal analysis of Lectin immunostained muscle fibre cross-sectional area (CSA) on GCM coronal sections of C57SOD1^{G93A} mice 129SvSOD1^{G93A} mice and relative NTG littermates (*n* = 6). For each section, the percent of fibre dimension was calculated relative to the total number of muscle fibres counted by Muscle J. Data are expressed as the mean (± SEM). Significance was calculated with 2-way ANOVA with uncorrected Fisher's LSD post-analysis (**P* ≤ 0.05; ****P* ≤ 0.001; *****P* ≤ 0.0001). (**E** and **H**) Representative confocal micrograph and longitudinal analysis of fibre type composition on GCM coronal sections of C57SOD1^{G93A} mice 129SvSOD1^{G93A} mice and NTG littermates (*n* = 6). A mix of antibodies against I (red), IIA (green), IIB (blue), MyHCs and Lectin (white) were used; IIX muscle fibres are unstained (black). Percent of fibre types was calculated relative to the total number of muscle fibres counted by Muscle J. Data are expressed as the mean (± SEM). Significance was calculated with Two-way ANOVA with uncorrected Fisher's LSD post-analysis (***P* ≤ 0.01).

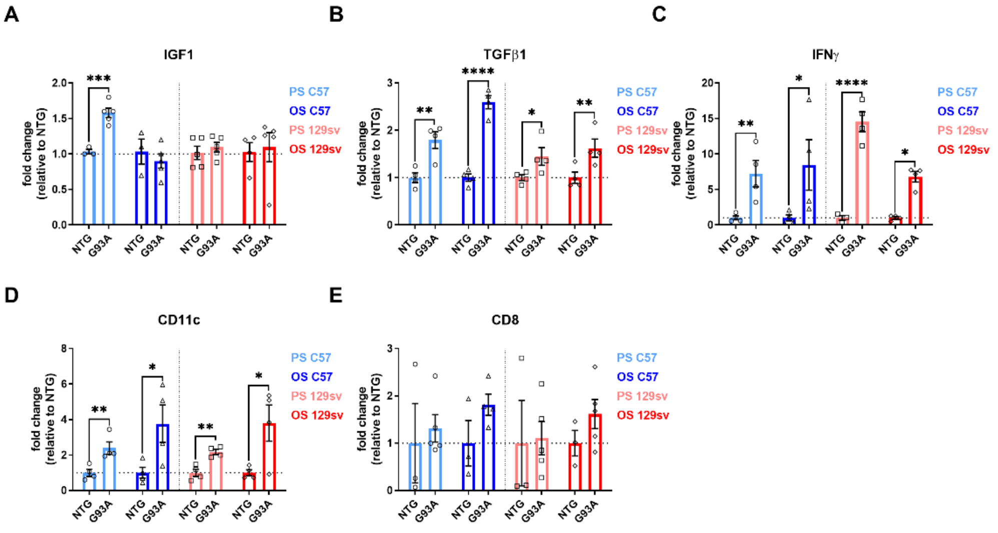

Supplementary Fig. 5. Muscle inflammatory signals in fast and slow progressing mice. (**A**-**D**) Real-time qPCR for IGF1 (**A**), TGFβ1 (**B**), IFNγ (**C**), CD11c (**D**) and CD8 (**E**) mRNA transcripts in GCM muscle of C57SOD1^{G93A} and 129SvSOD1^{G93A} mice compared with NTG littermates (*n* = 4). Data are expressed as the mean (± SEM). Significance was calculated with Two-way ANOVA with uncorrected Fisher's LSD post-analysis (**P* ≤ 0.05; ***P* ≤ 0.01; ****P* ≤ 0.001; *****P* ≤ 0.0001).
