# Supplementary figures and images for: Immune-mediated myogenesis and acetylcholine receptor clustering promote a slow disease progression in ALS mouse models
Source: Inflamm Regen. 2023 Mar 9;43:19. doi: 10.1186/s41232-023-00270-w (PMC9996869; doi:10.1186/s41232-023-00270-w)

**Fig. 3**

**b**

Agrin

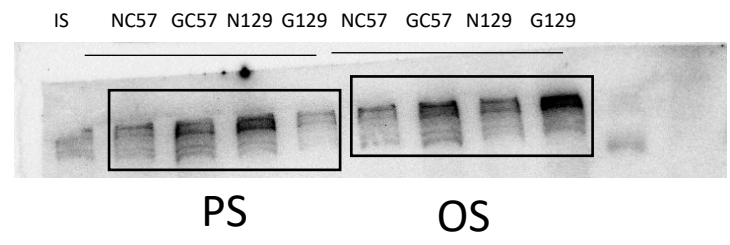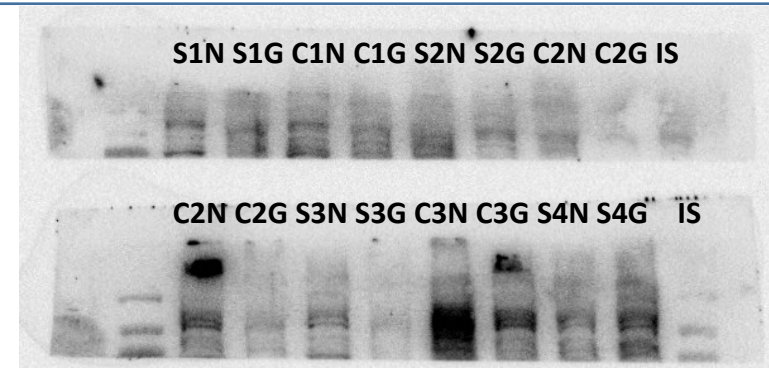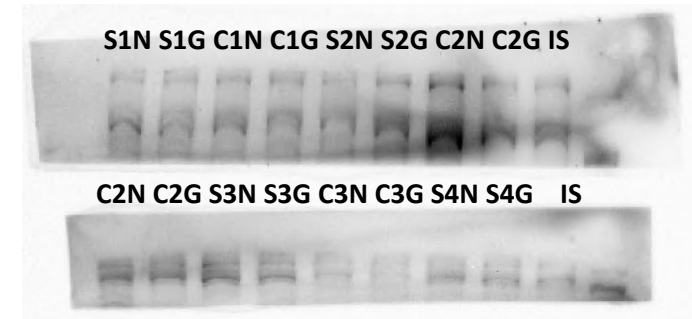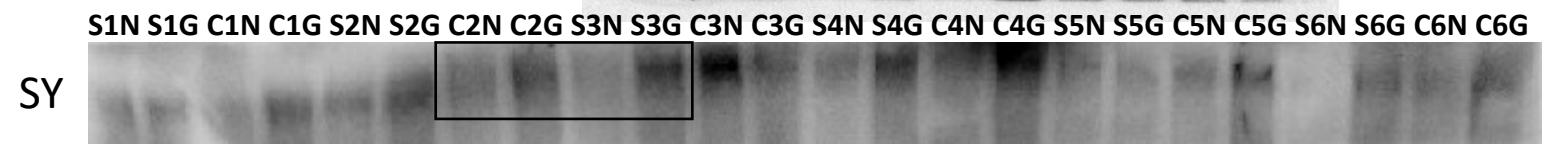

**c**

Dok-7

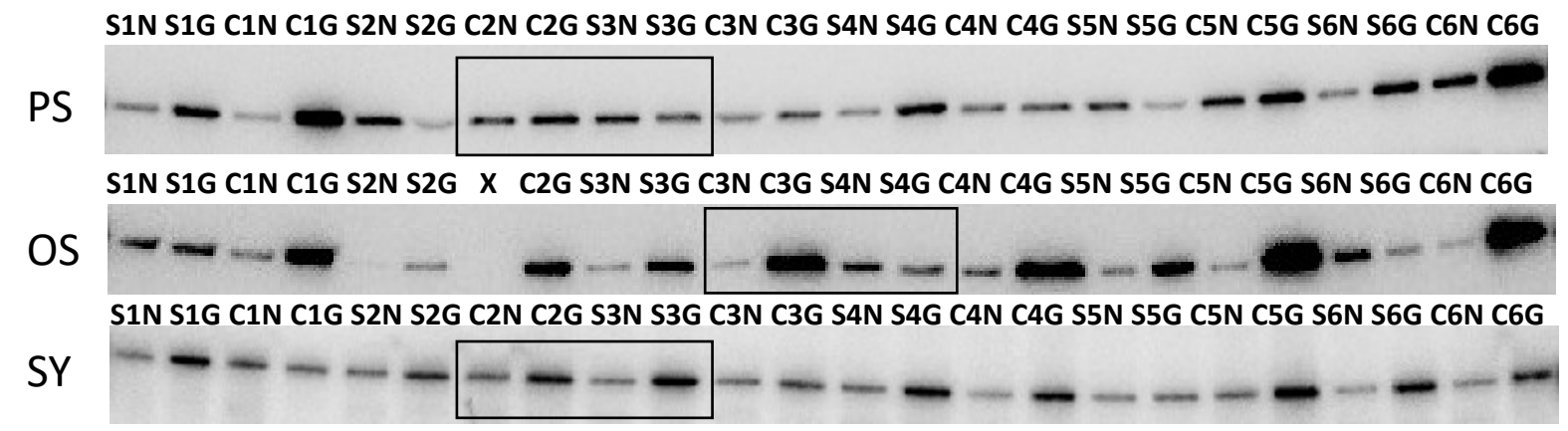

**d**

Rapsyn

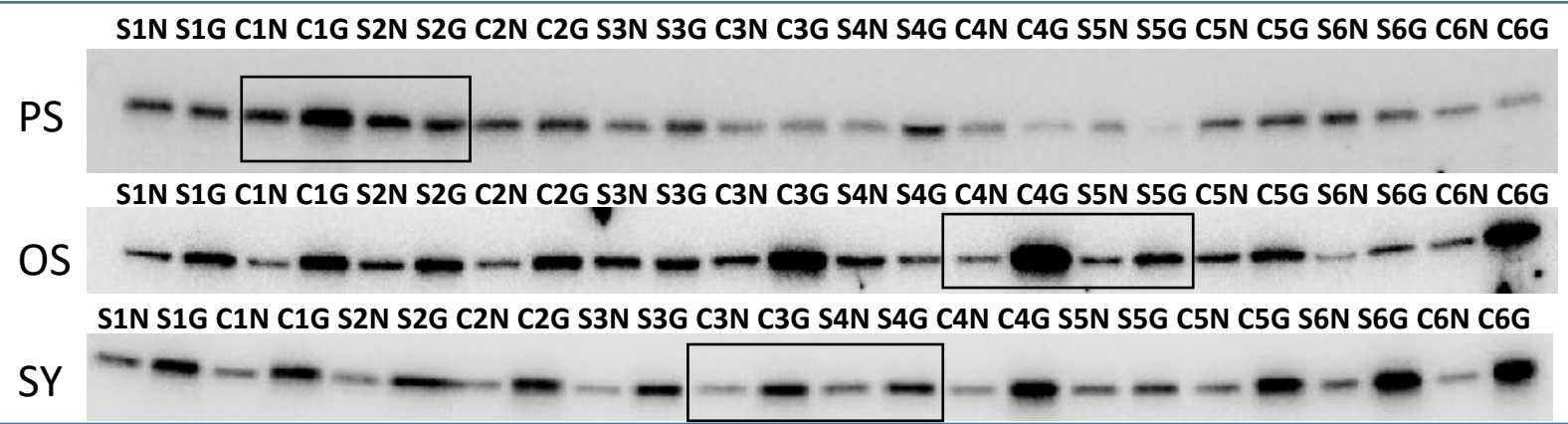

### Fig. 5

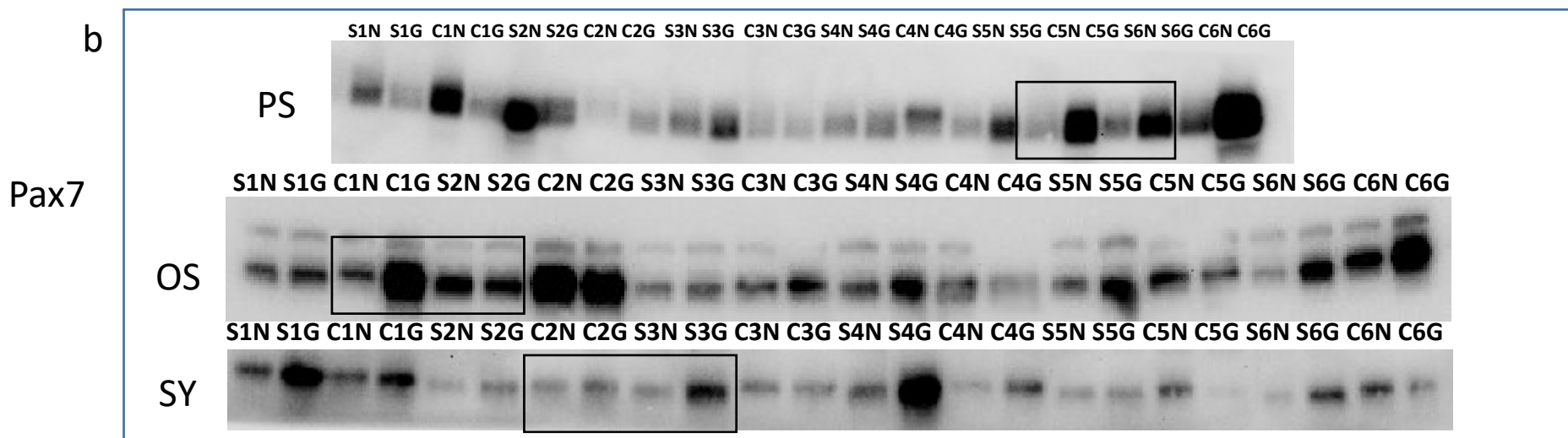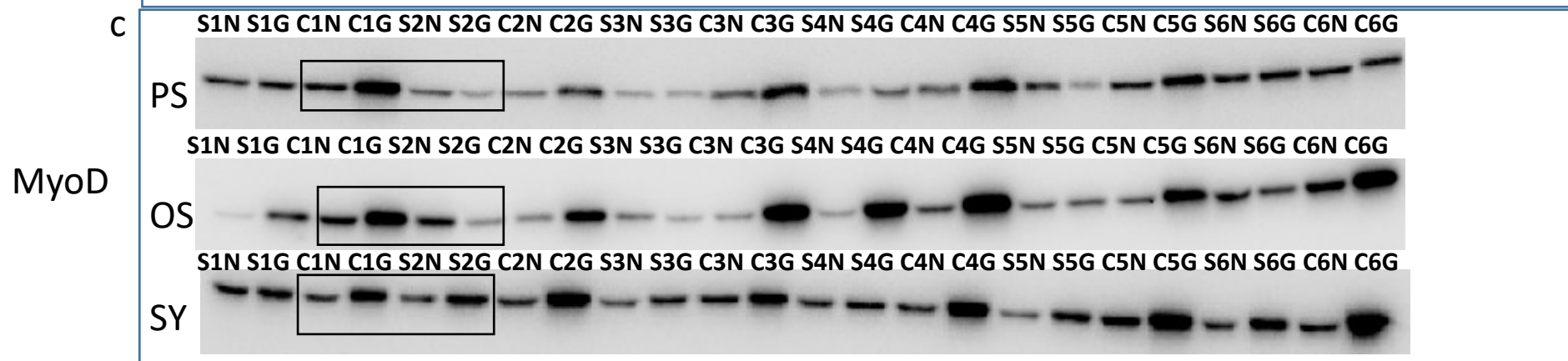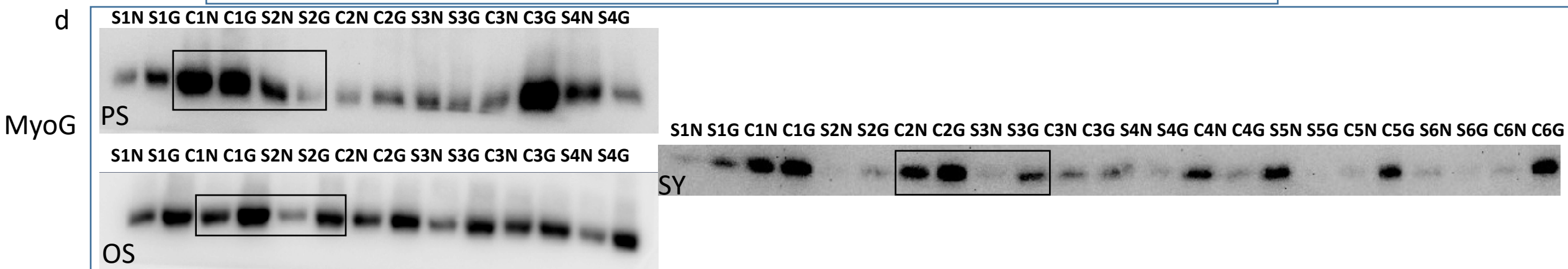

**Fig. 7** e

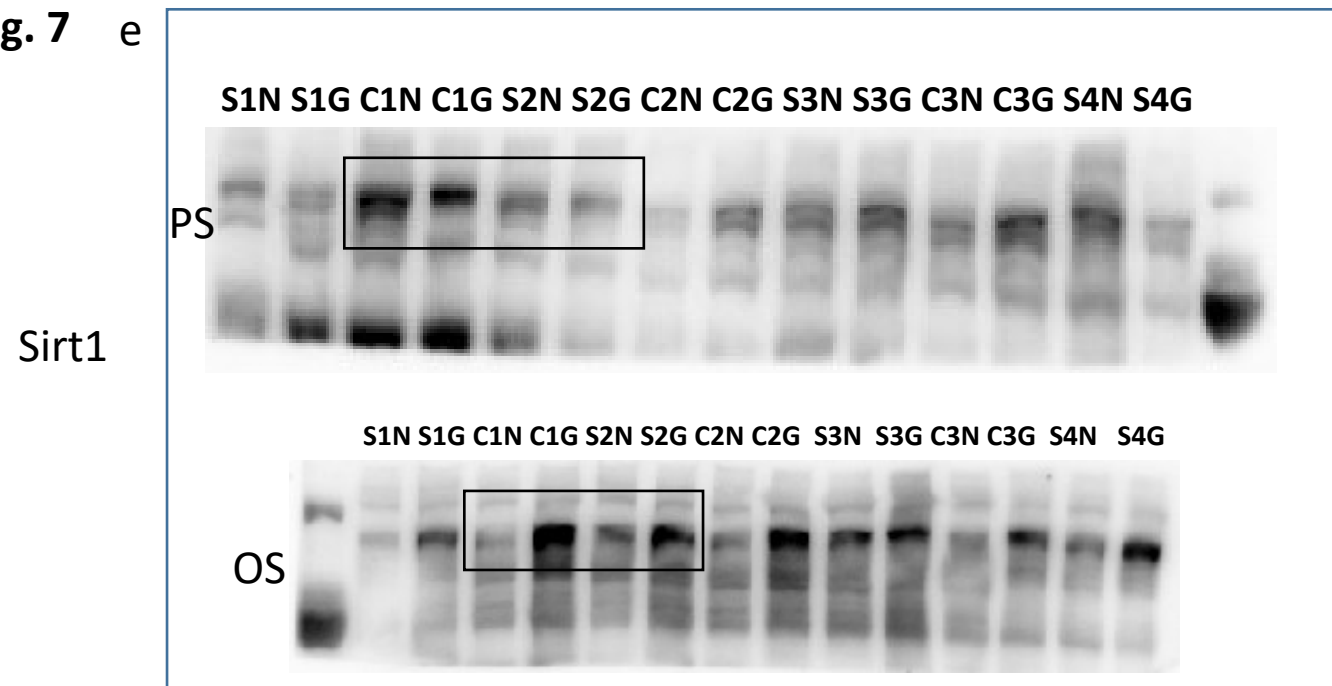

**f**

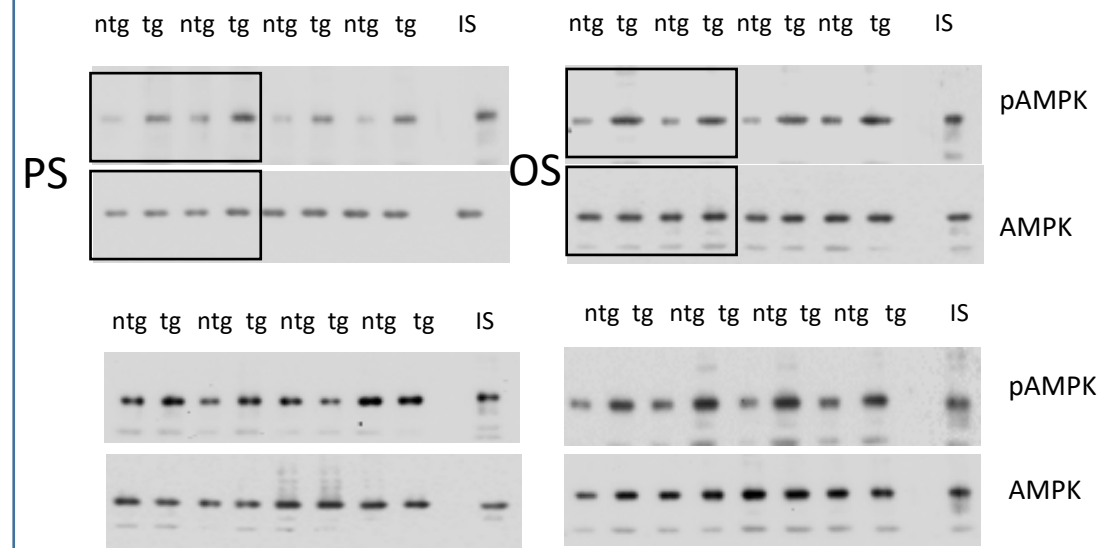

**g**

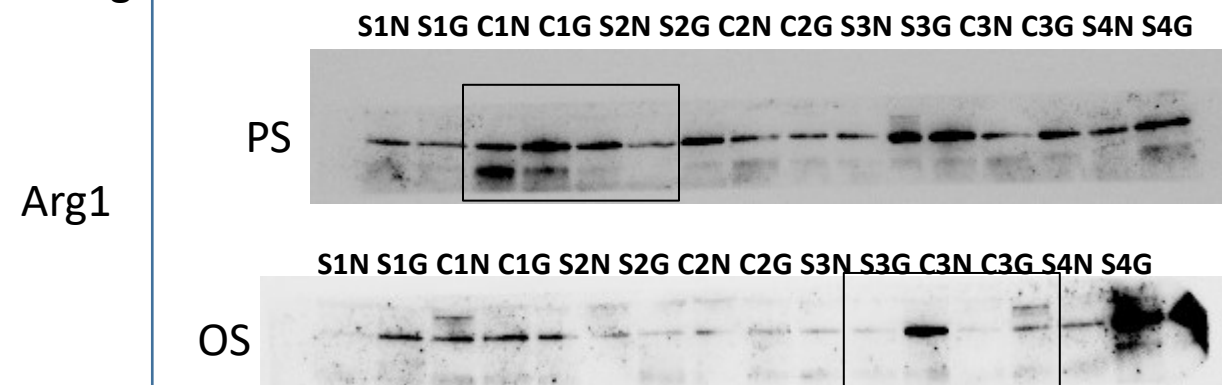

Fig. 8

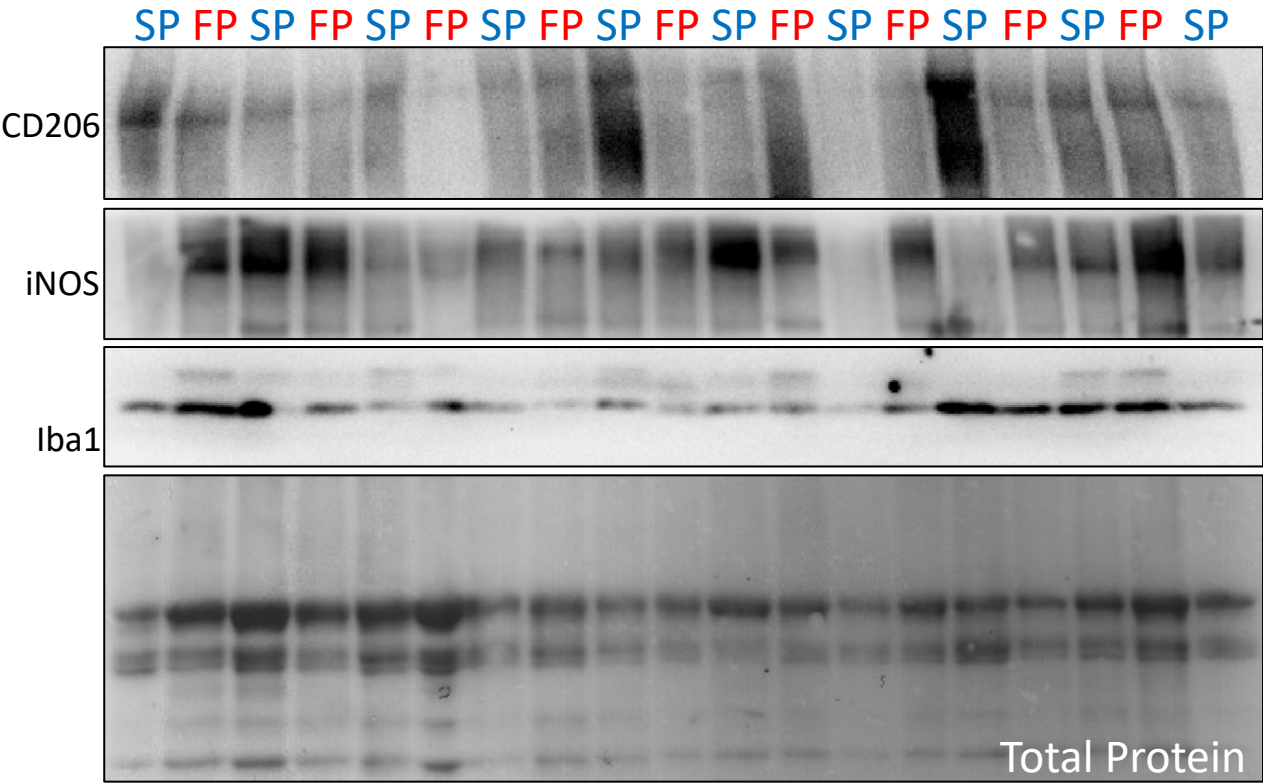

SP = SLOW PROGRESSING

FP = FAST PROGRESSING

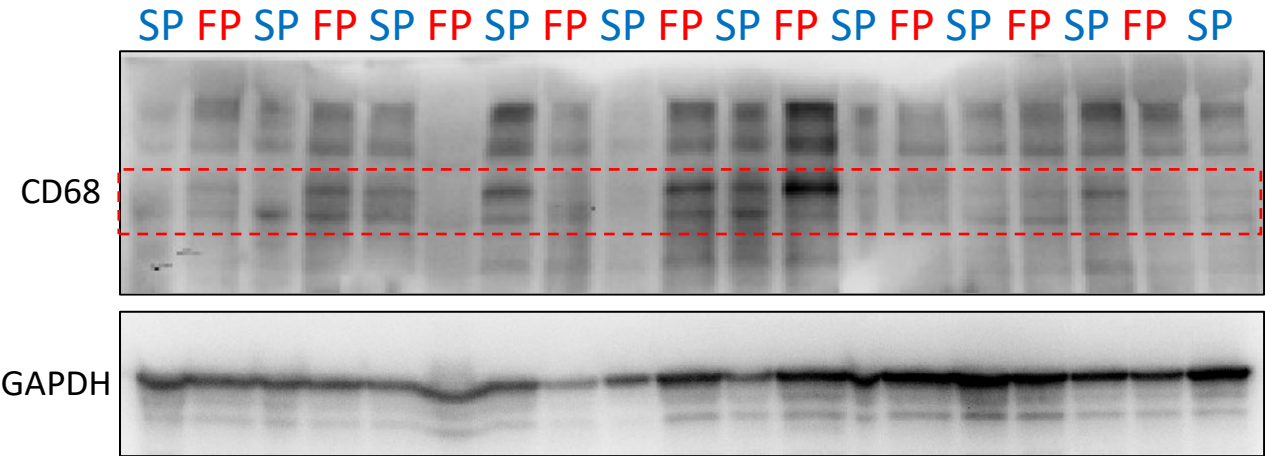

Supplement: Supplementary file 2 — Additional file 2. Original full-length blot images for Figs. 3A, 5A, 7D and 8A-D. [file 41232_2023_270_MOESM2_ESM.pdf]
